# Resistance potential of soil bacterial communities along a biodiversity gradient in forest ecosystems

**DOI:** 10.1002/mlf2.12042

**Published:** 2022-11-03

**Authors:** Jialiang Kuang, Dongmei Deng, Shun Han, Colin T. Bates, Daliang Ning, Wensheng Shu, Jizhong Zhou

**Affiliations:** ^1^ The Key Lab of Pollution Control and Ecosystem Restoration in Industry Clusters, Ministry of Education School of Environment and Energy, South China University of Technology Guangzhou China; ^2^ Institute for Environmental Genomics, and Department of Microbiology and Plant Biology University of Oklahoma Norman Oklahoma USA; ^3^ State Key Laboratory of Biocontrol, Guangdong Key Laboratory of Plant Resources and Conservation of Guangdong Higher Education Institutes College of Ecology and Evolution, Sun Yat‐sen University Guangzhou China; ^4^ Guangxi Key Laboratory of Green Processing of Sugar Resources College of Biological and Chemical Engineering, Guangxi University of Science and Technology Liuzhou China; ^5^ School of Life Sciences South China Normal University Guangzhou China; ^6^ School of Civil Engineering and Environmental Sciences University of Oklahoma Norman Oklahoma USA; ^7^ School of Computer Sciences University of Oklahoma Norman Oklahoma USA; ^8^ Earth and Environmental Sciences Lawrence Berkeley National Laboratory Berkeley California USA

**Keywords:** ecological resistance, forest soil bacterial community, latitudinal gradient, specialist–generalist balance, species richness

## Abstract

Higher biodiversity is often assumed to be a more desirable scenario for maintaining the functioning of ecosystems, but whether species‐richer communities are also more disturbance‐tolerant remains controversial. In this study, we investigated the bacterial communities based on 472 soil samples from 28 forests across China with associated edaphic and climatic properties. We developed two indexes (i.e., community mean tolerance breadth [CMTB] and community mean response asynchrony [CMRA]) to explore the relationship between diversity and community resistance potential. Moreover, we examined this resistance potential along the climatic and latitudinal gradients. We revealed that CMTB was significantly and negatively related to species richness, resulting from the changes in balance between relative abundances of putative specialists and generalists. In comparison, we found a unimodal relationship between CMRA and richness, suggesting that higher biodiversity might not always lead to higher community resistance. Moreover, our results showed differential local patterns along latitude. In particular, local patterns in the northern region mainly followed general relationships rather than those for the southern forests, which may be attributed to the differences in annual means and annual variations of climate conditions. Our findings highlight that the community resistance potential depends on the composition of diverse species with differential environmental tolerance and responses. This study provides a new, testable evaluation by considering tolerance breadth and response asynchrony at the community level, which will be helpful in assessing the influence of disturbance under rapid shifts in biodiversity and species composition as a result of global environmental change.

## Impact statement

The frequency and magnitude of extreme weather events and anthropogenic environmental changes have increased substantially. As a fundamental estimation of ecosystem functioning, biodiversity has long fascinated ecologists. However, whether higher biodiversity reflects higher tolerance is disputed. Here, we proposed two testable indexes by considering the species' environmental tolerance range and the asynchrony of their environmental responses. Our results suggested that the community resistance potential is not linearly related to the richness in species‐rich forest ecosystems. Instead, how coexisting species respond differentially to environmental changes may play a more important role in predicting the stability of community composition and ecosystem functioning under disturbance.

## INTRODUCTION

Extraordinarily diverse below‐ground microbial communities drive the multifunctionality in terrestrial ecosystems^1^, ^2^. Accumulating evidence has revealed a positive relationship between microbial biodiversity and ecosystem functioning^2^, ^3^, ^4^, ^5^. Current studies in microbial ecology often assume that high diversity is, implicitly, a more desirable scenario for maintaining the functioning of ecosystems. Whether communities with higher diversity are more resistant to disturbance remains controversial^6^, ^7^, ^8^, ^9^, ^10^. A larger number of coexisting species are expected to provide a greater buffering effect to insure ecosystems against declines in their functioning when different species respond differently to environmental changes^11^, ^12^, ^13^. However, more diverse microbial communities might not necessarily be composed of more distinct members that are capable of more differential responses. Hence, microbial diversity and the differential responses of species within the entire community are relevant for a large‐scale assessment of ecological resistance to rapid global changes. However, it remains largely unexplored, especially in species‐rich soil ecosystems.

**Figure 1:**
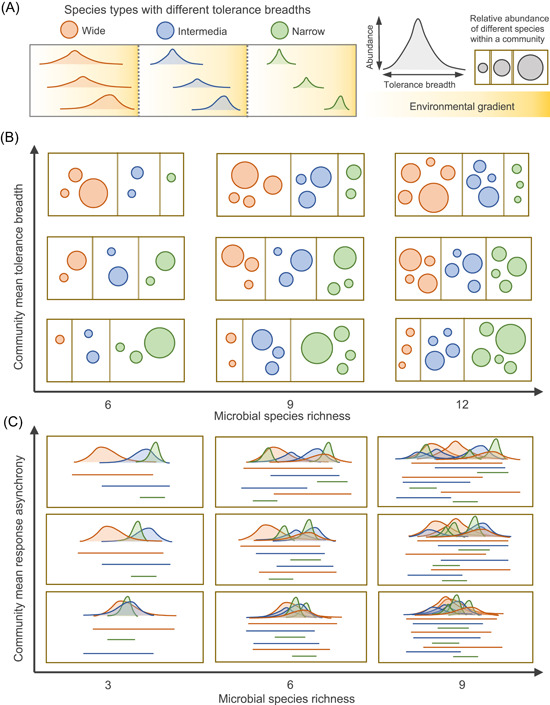
Conceptual diagrams showing different levels of community mean tolerance breadth (CMTB) and community mean response asynchrony (CMRA) along the gradient of microbial diversity. (A) Different species types have different tolerance breadths along the environmental gradient. (B) Communities with different species richness can result in a similar level of CMTB (horizontal panels) and the CMTB varies among communities with consistent biodiversity (vertical panels), suggesting that the relationship between biodiversity and CMTB depends on the community structure (e.g., relative abundance and composition) of different species with different ranges of environmental tolerance. (C) Similar to CMTB, more diverse communities might not necessarily be composed of species with more distinct environmental optima and less overlapped tolerance breadth, suggesting that the relationship between biodiversity and CMRA may vary among different ecosystems.

Different species respond to climate changes at different rates and to varying degrees^14^. The average magnitude of environmental tolerance range^15^ and the asynchrony^16^ of species responses to a perturbation can substantially affect the strength of the buffering effect^13^. The environmental tolerance range partly defines an organism's niche (i.e., tolerance breadth), describing the set of conditions under which it can inhabit^15^, ^17^ (Figure 1A). Some microbial taxa that can tolerate a wide range of environmental conditions (“generalists”) are more likely to be ubiquitous; in contrast, other taxa with a narrow tolerance breadth (“specialists”) can only persist under more specific conditions^18^. Given that global climate changes can produce abiotic forces or constraints as environmental filters to limit the type of species with different tolerance breadth^9^, niche‐based modeling has been used to improve the prediction of species distributions under climate change scenarios^19^, ^20^, ^21^. Logically, the tolerance breadth can be extended to the community level as the mean tolerance breadth of species present in that community, reflecting community response to the environmental disturbance^22^. Although a community with higher community mean tolerance breadth (CMTB) is inferred to be more resistant to environmental disturbances, the relationship between biodiversity and CMTB depends on the community structure (e.g., relative abundance and composition) in terms of generalists/specialists (Figure 1B). Therefore, the compositional dynamics of generalists and specialists in the face of perturbations can also account for the persistence of microbially driven ecosystem services^23^.

Alternatively, temporal and spatial synchrony have been widely studied to examine the correlated fluctuations of population dynamics over time and across geographically distant locations^24^, ^25^, ^26^, ^27^, ^28^, which can play a significant role in ecosystem stability^13^, ^29^, ^30^. From the perspective of ecological resistance, asynchrony of species' responses to perturbations describes the niche differentiation of environmental tolerance (i.e., how different species respond differently to variations in their environment)^31^. If ecologically similar species in a community have more diverse environmental optima and less overlapped tolerance breadth along the environmental gradient, these asynchronous populations may compensate for each other when they encounter a disturbance and therefore limit the influence^26^, ^32^. In contrast, high synchronization suggests that different species respond similarly to environmental changes. In an extreme case, when there is no asynchrony in the species responses, the entire community behaves like a single species^13^. Thus, higher community mean response asynchrony (CMRA) may enhance ecological resistance; yet, the relationship between biodiversity and CMRA may vary among different ecosystems (Figure 1C).

Collectively, the microbial biodiversity and the composition of diverse species with differential environmental tolerance and responses determine the magnitude of CMTB and CMRA. Communities with higher degrees of CMTB and/or CMRA may suffer less from a particular environmental change or extreme event with a lower risk of disruption for microbially driven ecosystem functions and are thus considered more resistant. Here, we present a study examining the large‐scale patterns of CMTB/CMRA and their relationships with richness among forest soil bacterial communities in China. Our goal was to refine and assess the potential of community resistance to environmental changes along a biodiversity gradient across space.

## RESULTS

### Large environmental gradient among forest ecosystems

**Figure 2:**
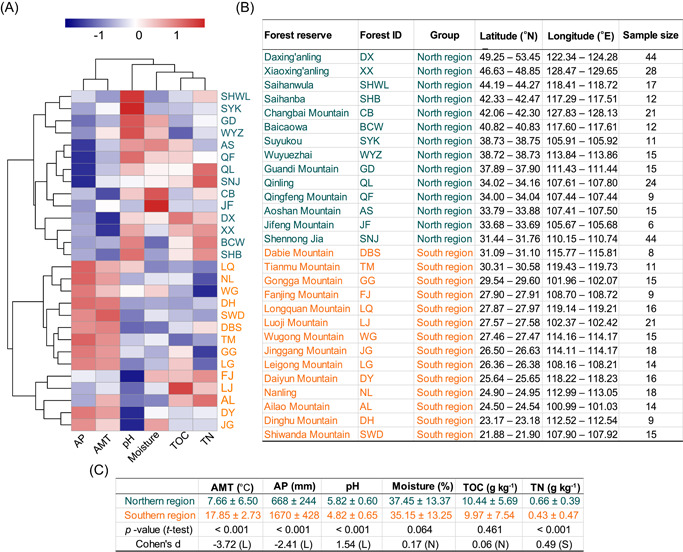
Spatial distribution and the differences in the environmental properties of the 28 forest reserves. In this study, a total of 472 soil samples were used to represent the soil bacterial communities and environmental properties from 28 natural forest reserves across China. (A) The 28 forest reserves were mainly divided into two groups (i.e., the northern and southern regions) based on the six environmental variables by hierarchical cluster analysis. (B) The sample sizes and the location ranges of soil samples in each natural forest reserve are shown and ordered by latitudes. (C) The environmental conditions between these two regions were compared using a *t*‐test. The magnitude of the differences was estimated by Cohen's *d* effect size: N (negligible, |*d*| < 0.2), S (small, 0.2 ≤ |*d*| < 0.5), M (medium, 0.5 ≤ |*d*| < 0.8), and L (large, |*d*| ≥ 0.8). Negative values of Cohen's *d* effect size indicate higher mean values of environmental conditions for soil samples from the southern region. Detailed environmental conditions are shown in Table S1.

Our soil samples from 28 natural forest reserves were characterized by a wide variety of annual mean temperature (AMT) (−5.69°C to –22.76°C) and annual precipitation (AP) (100–2214mm), as well as soil pH (3.67–7.05), moisture (5.74%–68.14%), and the contents of total organic carbon (TOC) (1.16–42.63 g kg^−1^) and total nitrogen (TN) (0.01–3.78 g kg^−1^) (Table S1). These forest ecosystems were mainly clustered into two groups (i.e., northern and southern regions) with significant differences in environmental conditions (PERMANOVA test, *Df* = 471, *F* = 176.32, *p* < 0.001; Figure 2). In particular, forests from the southern region were characterized by significantly (*p* < 0.001) higher AP and AMT and lower soil pH and TN content than those found in the northern region with non‐negligible effect sizes (Figure 2). In general, the investigated forest ecosystems across China revealed a large environmental gradient and captured the environmental tolerance/preference of soil bacterial organisms that they harbored and were suitable for addressing our questions in this study.

### Bacterial diversity and their tolerance breadth

The bacterial species richness of our soil samples was significantly and unimodally related to latitudes and peaked at around 35°N–40°N (*R* ^2^ = 0.385, *p* < 0.001, quadratic regression model) (Figure S1), which is the boundary between southern and northern regions (i.e., subtropical and temperate zones) with distinct environmental conditions (Figure 2). Among all samples, the most dominant lineages were *Acidobacteriota*, *Gammaproteobacteria*, *Alphaproteobacteria*, *Actinobacteriota*, and *Verrucomicrobiota*, accounting for 23.5%, 17.3%, 15%, 12.5%, and 9.6% of the relative abundances, respectively. Some other phyla were less abundant, but still detected in most of the samples, including *Planctomycetota* (4.8%), *Chloroflexi* (4.1%), *Bacteroidota* (3.8%), *Firmicutes* (1.4%), *Gemmatimonadota* (1.3%), and *Myxococcota* (1.3%). The tolerance breadth (TB) of the operational taxonomic units (OTUs) was calculated to estimate their environmental adaptation, and a given bacterial OTU with a larger TB value was considered as a putative “generalist”. Our results showed that both environmental “generalist” and “specialist” were broadly distributed across the phylogenetic tree (Figure S2), implying that the habitat range of bacteria was phylogeny‐independent among our study sites.

### Method independence of CMTB and CMRA

**Figure 3:**
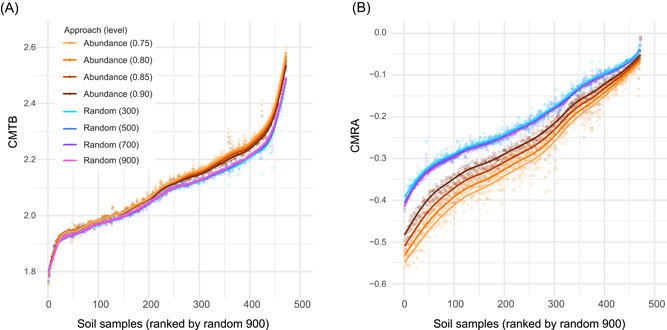
Comparison of indexes of the 472 soil samples that were calculated by different approaches of bacterial OTUs' selection at different levels. (A) CMTB. (B) CMRA. For the approach based on a consistent abundance of bacterial communities, the bacterial OTUs of a sample were ranked by their relative abundances in descending order. The most abundant OTUs, representing the accumulated abundances at 0.75, 0.80, 0.85, and 0.9, were retained for the index calculation. For the approach based on a consistent number of bacterial richness, 300, 500, 700, and 900 bacterial OTUs of a sample were randomly selected and the OTUs' selection at each level was repeated 100 times. For each level, the indexes were calculated as the average values of these 100 sets of bacterial OTUs. OTU, operational taxonomic unit. CMRA, community mean response asynchrony; CMTB, community mean tolerance breadth.

To test if the two indexes of CMTA and CMRA were comparable features among samples, they were calculated based on the bacterial OTUs that were selected using two approaches at four different levels. We found that the CMTB (or CMRA) values were strongly and significantly related irrespective of the approaches and levels that we used (Pearson *r* > 0.96, *p* < 0.001, Figure 3 and Table S2). These results indicated that the patterns of CMTB and CMRA among samples were method‐independent and reflected important features of bacterial communities. Therefore, we considered that the comparison of these indexes could be used to estimate the differential potential of community‐level resistance to environmental disturbances. We used the values based on the random selection of 900 bacterial OTUs for the subsequent analyses.

### Relationships between CMTB/CMRA and bacterial species richness

**Figure 4:**
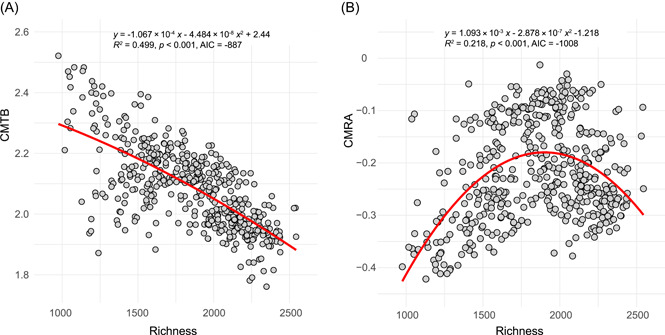
Relationships between bacterial species richness and different indexes. (A) CMTB. (B) CMRA. Linear and quadratic regressions were performed to examine these relationships. Quadratic regression models were selected owing to their lower values of the Akaike information criteria (AIC) index than those of linear models (CMTB: −887 vs. −868; CMRA: −1008 vs. −919; AIC values: quadratic vs. linear).

**Figure 5:**
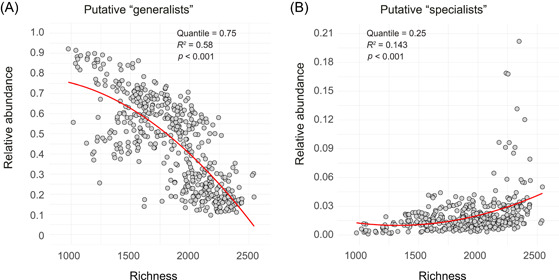
Relationships between bacterial species richness and the relative abundances of different microbial groups. (A) Putative “generalists.” (B) Putative “specialists.” Putative “generalists” and “specialists” were defined based on the distribution of TB values among bacterial OTUs at five quantiles (“generalists”: 0.95, 0.90, 0.85, 0.80, and 0.75; “specialists”: 0.05, 0.10, 0.15, 0.20, and 0.25). Similar patterns for putative “generalists” at quantiles of 0.95, 0.90, 0.85, and 0.80 as well as for putative “specialists” at quantiles of 0.05, 0.10, 0.15, and 0.20 are shown in Figure S2. Quadratic regression models were selected owing to their lower values of the Akaike information criteria (AIC) index than those of linear models. The distribution of TB values is shown in Figure S1. OTU, operational taxonomic unit; TB, tolerance breadth.

We observed that the CMTB was significantly and negatively (*R* ^2^ = 0.499, *p* < 0.001) related to the bacterial species richness (Figure 4A). In addition, our results revealed a significant (*p* < 0.001) decrease in the relative abundance of putative “generalists” along with an increase in microbial diversity (Figures 5A, S3 and S4A‐D). In contrast, we found an opposite trend for the relative abundance of putative “specialists” (Figures 5B and S4E‐H). These results indicated that bacterial communities with higher richness were composed of a larger proportion of putative “specialists” but less putative “generalists,” resulting in significantly lower values of CMTB.

**Figure 6:**
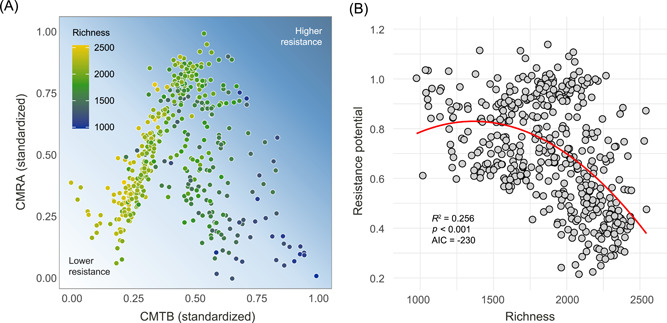
Relationships between species richness and resistance. (A) Distributions of CMTB/CMRA among 472 soil microbial communities. (B) Resistance potential along the gradient of bacterial species richness. Values of CMTB and CMRA were standardized with a range from 0 to 1. Resistance potential was calculated as the square root of the sum of ${\mathrm{CMTB}}_{\mathrm{stand}}^{2}$ and ${\text{CMRA}}_{\mathrm{stand}}^{2}$. Communities with higher degrees of both CMTB and CMRA (i.e., greater resistance potential) were expected to be more resistant to environmental changes.

Compared to CMTB, we found a hump‐shaped relationship between CMRA and richness (*R* ^2^ = 0.218, *p* < 0.001) (Figure 4B). This pattern suggested that bacterial species in communities at a moderate biodiversity level had larger differences in environmental optima and a greater degree of response asynchrony. Together, by considering the patterns of both indexes along the biodiversity gradient (Figure 6A), we found a nonlinear relationship (*R* ^2^ = 0.256, *p* < 0.001) between richness and the resistance potential (Figure 6B), revealing that bacterial communities at a moderate biodiversity level tend to experience less influence from a disturbance.

### Resistance potential along climatic and spatial gradients

**Figure 7:**
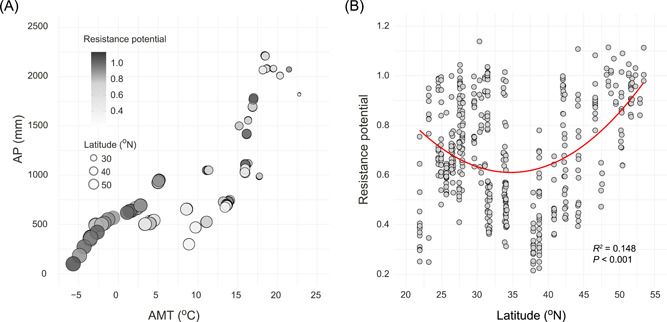
Resistance potential along climatic and spatial gradients. (A) Relating resistance potential to annual precipitation (AP) and annual mean temperature (AMT). (B) Spatial distribution of resistance potential along latitudes.

Random forest analysis revealed that climate conditions (i.e., AMT and AP) were more important predictors of resistance potential than edaphic variables (Figure S5). Because AMT and AP were also strongly related to latitude (Figure S6), we visualized the distribution pattern of resistance potential along the climatic and spatial gradients (Figure 7A). We found a significant (*R* ^2^ = 0.148, *p* < 0.001) U‐shaped distribution of resistance potential along the latitudinal gradient (Figure 7B). This pattern revealed that the values of resistance potential were relatively low in environments under moderate temperature and precipitation levels (Figure 7A), with minimum values around 35°N–40°N (Figure 7B).

### Generality of resistance patterns at a local scale

Given that the climate conditions, including annual means (Figure 2) and annual variation (Figure S7), were distinct between the southern and northern regions in China, we further explored the generality of our observed patterns across different forest ecosystems along the latitudinal gradient. Our results revealed that the overall relationships of richness–CMTB (Figure 4A, negative) and richness–CMRA (Figure 4B, hump‐shaped) were more commonly found in the northern region (Figures S8 and S9). In comparison, in the southern region, we observed various relationships of richness–CMTB (Figure S8) but a consistent positive correlation of richness–CMRA (Figure S9) across different forests. The distinct climate conditions between the northern and southern regions reflect differential degrees of environmental stress and can act as strong forces regulating specialist–generalist balance and species interactions, thereby resulting in these observed local differential patterns.

## DISCUSSION

Global climate changes are causing shifts in the richness, distribution range, and composition of species worldwide and affecting ecological stability and the sustainability of ecosystem functions^14^. How environmental disturbance influences microbial biodiversity has been broadly addressed^33^, ^34^, ^35^. Nevertheless, how biodiversity reflects the potential of community‐level resistance is far from clear. We argue that the resistance potential depends on the composition of diverse species with differential environmental tolerance and responses. In this study, we have defined CMTB and CMRA to estimate the community‐level tolerance breadth and responses asynchrony to perturbations, respectively. We have applied these indexes to explore the distribution pattern of resistance potential among species‐rich forest ecosystems along broad environmental and spatial gradients. By showing a clear unimodal pattern, our findings provide important insight into the controversy about the relationship between microbial biodiversity and community resistance to disturbance.

### Specialist–generalist balance and community tolerance

Environmental specialists and generalists, which are identified based on their tolerance and survivability^36^, ^37^, coexist in diverse habitats. However, their relative abundances (i.e., the specialist–generalist balance) vary markedly among environments^38^. In this study, we found that the tolerance breadth of bacterial communities was negatively associated with biodiversity, resulting from a decreasing ratio of generalists' to specialists' relative abundances when species richness increases (Figure S10). This result confirmed that high richness drives community‐level specialization^39^, highlighting that species‐richer communities can be composed of more members that are more specialized.

In addition, we found that the mean relative abundances of putative “specialists” were relatively higher than those of putative “generalists” (Figure S3B), which is likely because of their superior local adaptation (e.g., higher performance and niche preference) in the given environment^40^. However, compared with generalist microbes with persistence advantage, specialist species only adapt to a narrow range of environments^37^ and are disadvantaged in more drastic changing environments^41^. Due to the synergistic effects of a narrow niche and small range size, the populations of specialists may be more vulnerable and rapidly decrease or even become extinct when they are at risk of intense global changes^15^, ^42^.

In contrast, although specialists can outperform generalists owing to the higher efficiency of resource utilization under optimal conditions, habitat generalists are increasingly realized to be metabolically flexible and can become dominant in highly dynamic environments^36^. Thus, environmental disturbance and habitat heterogeneity are predicted to favor generalists^38^. As a result, communities with a higher proportion of generalists are expected to be more resistant to disturbances (Figure S11). Our findings suggest that the specialist–generalist balance plays an important role in the potential of community resistance to environmental disturbance.

### Response asynchrony and community resistance

Previous studies have shown that species asynchrony promotes ecological resistance in fluctuating environments and enhances community stability^43^, ^44^, ^45^, ^46^ due to the complementarity of ecologically distinct species with large differences of species‐specific responses to environmental fluctuations^26^, ^32^, ^47^. Our results revealed a hump‐shaped relationship between response asynchrony and bacterial species richness. Communities with lower richness were found to be composed of a larger proportion of putative environmental “generalists,” which distribute ubiquitously across various environments. Thus, a larger overlap of tolerance breadth in these “generalists”‐dominated communities possibly accounted for a lower response asynchrony.

As species richness increases, specialization is assumed to enable the coexistence of more species and support high diversity, resulting in a greater number of habitat‐specialized species (i.e., environmental specialists)^39^. In addition, to coexist, specialists tend to interact with generalists, and the increasing richness is suggested to result in an increase of extreme specialists^48^. As a result, a decreasing overlap of tolerance breadth is expected, leading to an increase of response asynchrony when richness increases.

However, our results showed that the response asynchrony peaked at a moderate diversity level and declined when the richness kept increasing. This pattern suggested that the continuous increase in species richness (mainly “specialists”) decreased the overall differences in their environmental optima. This finding implies that higher biodiversity might not always lead to higher community resistance, especially when different species have synchronous responses to environmental changes.

### Spatial pattern of resistance potential

Our results suggested that the resistance potential was non‐linearly correlated to latitude with minimum values around 35°N–40°N, implying that communities in this zone might be more sensitive to environmental changes. This region in China had experienced dramatic climate fluctuations during the last 20 ka BP (kiloyear Before Present)^49^ and is a transition area between subtropical and temperate biomes^50^ with a sharp shift in climate conditions (Figure 2). Although “Janzen's mountain passes hypothesis” assumes that tropical mountains have higher diversity due to the relatively stable climate regimes^51^, our results suggested that higher diversity could have resulted from more fluctuations in temperature in this mid‐latitude region (Figures S1 and S7A), which is expected in ecotone^52^. As shown above, communities with higher diversity were composed of a higher proportion of specialists (Figure S10) and were expected to be less resistant to disturbances in our studying forest ecosystems (Figure S11). Together, this observed pattern implied that the reduction of physiological tolerance may not necessarily limit the community biodiversity^53^, and higher diversity may not always lead to higher community resistance. However, the communities with a larger species pool might be more resilient because they are more likely to contain taxa with complementary response traits, which may facilitate ecosystems to recover without switching to a qualitatively different state^41^, ^54^.

### Differential local patterns along latitude

Our study captured wide gradients of climate conditions, including the annual means (Figure 2 and Table S1) and annual variations (Figure S7), possibly yielding the local differential patterns along latitude. Forests in the northern region are characterized by lower AMT and AP but higher annual variations than those in the southern region, representing relatively harsh, fluctuating environments for the growth of microorganisms. Under these relatively stressful conditions, generalists with persistence advantage and higher metabolic flexibility are more likely to competitively exclude habitat‐specialized species^36^, leading to a decrease in biodiversity. Furthermore, the dominance of generalists in species‐poor communities may result in the more apparent negative correlations between species richness and CMTB (Figure S8, negative relationship in the northern region).

Moreover, the distinct climate conditions between the northern and southern regions influence the outcome of the interaction among co‐occurring species. The stress gradient hypothesis (SGH) suggests that positive interactions should be more prevalent in stressful environments, while permissive environments should favor competition^55^, ^56^. In this study, forests in the northern region were relatively stressful and had fluctuating environments in terms of climate conditions, possibly promoting positive interactions among species. This may allow different species to coexist even though they have similar ecological traits with synchronous environmental responses (Figure S9, hump‐shaped relationship in the northern region). In contrast, according to the SGH, intensive competition is expected in communities in the southern region under relatively moderate and stable conditions, which can drive specialization^39^, ^57^. As a result, species‐rich communities in southern region likely force co‐occurring species to have narrower niches and more specified traits, leading to the positive relationship between response asynchrony and richness (Figure S9, positive relationship in the southern region).

In summary, we propose two testable indexes for the evaluation of community resistance potential, which is regulated by the shifts in species composition. Our results have shown how the geographic pattern of community resistance is related to the biodiversity along the climatic and latitudinal gradients. Global changes, such as warming and eutrophication, have been demonstrated to mediate the microbial diversity, composition, functional traits, and species co‐occurrence networks^35^, ^58^, ^59^, ^60^, which are also expected to affect the community resistance potential following the mechanisms that we have described. Future work is needed to predict how the long‐term effects of such environmental change influence the community resistance potential by mediating the temporal dynamics of communities and consequently impact ecosystem functioning. We believe that our work can inspire further analyses of the spatiotemporal patterns of community resistance, which can deepen our understanding of the relationship between biodiversity and ecosystem stability.

## MATERIALS AND METHODS

### Study sites, environmental properties, and soil bacterial communities

We collected a total of 472 soil samples from 28 forest reserves located across a broad range of latitudes (21.88°N–53.45°N) and longitudes (100.01°E–129.65°E) in China in 2012 (Figure 2 and Table S1). The detailed information about soil sampling, soil property measurement, climate conditions, and the molecular characterization of soil bacterial communities has been previously described^50^, ^61^, ^62^. In brief, top layer soils (0–10 cm in depth) were collected from 3 to 14 sites in each forest reserve. The numbers of sites were determined approximately based on the sizes of forest reserves. We selected the locations of sites to represent the gradient of elevation for each forest reserve and recorded the coordinates using a portable GPS machine. In each sampling site, three soil samples were collected as biological replicates that were located about 5 m apart from each other. After the removal of vegetation litter, soils were sampled and stored in sterilized polyethylene bags and kept on ice immediately in the field, and then transported to the laboratory. We downloaded the daily records of temperature (°C) and precipitation (mm) from 194 weather stations in the National Meteorological Information Center (http://data.cma.cn/en). In this study, we used the data from 2012 to represent the climate conditions. For temperature, we calculated the monthly means based on the daily data and then averaged the 12 monthly means as the AMT. For precipitation, we summed up the daily data to generate the monthly sums and then added up the 12 monthly sums as the AP. We estimated the AMT and AP for each site by applying the kriging interpolation method^63^ using the “gstat” package in R and obtained the interpolated AMT and AP according to the site location.

To assess the bacterial environmental tolerance range in this study, we used four major variables of soil properties, including pH, moisture, TOC, and TN, as well as two variables of climate conditions, including AMT and AP (Table S1).

To characterize the soil bacterial communities, we amplified and sequenced the V4 hypervariable region of the 16S rRNA gene using the 515F (5'‐GTGCCAGCMGCCGCGGTAA‐3') and 806R (5'‐GGACTACHVGGGTWTCTAAT‐3') primer pair. We clustered the qualified reads into OTUs at a 97% similarity threshold and rarefied 9736 bacterial reads for each soil sample. We aligned the representative bacterial OTUs using Mafft^64^ and constructed the phylogenetic tree using FastTree^65^. Taxonomic classification of the representative bacterial OTUs was determined based on the pre‐trained Silva 138 Naive Bayes 515F/806R classifier^66^. The display and the annotation of phylogenetic tree were performed by iTOL^67^. The details of DNA extraction, library construction, sequencing, and data processing have been described previously^50^, ^61^, ^62^. The representative sequences of bacterial OTUs and their abundances in each sample are available in figshare (https://doi.org/10.6084/m9.figshare.17711495).

### CMTB

We considered a given OTU as a putative environmental “generalist” if it occurs across habitats with distinct conditions. In this study, we used the measure of multivariate dispersion^68^, ^69^ based on the standardized data of six edaphic and climatic variables to estimate the difference of environments where a given OTU inhabits. A given bacterial OTU may occur in a wide range of habitats; yet, its abundance may vary among different samples under different environmental conditions. It is recognized that generalists with a larger tolerance breadth distribute more evenly across various environments, while specialists with a smaller tolerance breadth are only dominant in specific habitats^70^. Therefore, we calculated the tolerance breadth of a certain bacterial OTU *m* (TB_*m*_) as the average distance of samples (i.e., environments where the target OTU is detected) to the centroid weighted by the relative abundances of OTU *m* in different samples according to Equations 1 and 2 (*fdisp* function, “FD” package in R)^69^.

For a target OTU *m*, the weighted centroid was calculated following Equation 1.

(1) $$C=\left[c_{i}\right]=\frac{\sum a_{j}x_{\mathrm{ij}}}{\sum a_{j}}$$

where *C* is the weighted centroid in the *i*‐dimensional space, *i* is the number of environmental variables, *a*_*j*_ is the abundance (i.e., number of sequencing reads) of OTU *m* in sample *j*, and *x*_*ij*_ is the standardized value of environmental variable *i* in sample *j*. We then computed the TB_*m*_ as the weighted mean distance *Z* to the weighted centroid *C* according to Equation 2.

(2) $${\mathrm{TB}}_{m}=Z=\frac{\sum a_{j}z_{j}}{\sum a_{j}}$$

where *a*_*j*_ is the abundance of OTU *m* in sample *j* and *z*_*j*_ is the distance of sample *j* to the weighted centroid *C*.

A larger value of TB suggests that the bacterial OTU occurs across more distinct environments and is considered as a putative “generalist.” This index of multivariate dispersion has no upper limit and is unaffected by the number of samples (i.e., environments where OTU *m* is detected). More importantly, it can be computed from any distance or dissimilarity measure, it can handle any number and type of environmental variable (e.g., quantitative and categorical data), and it is not strongly influenced by outliers^68^, ^69^.

We estimated the CMTB by calculating the abundance‐weighted mean of TB of bacterial OTUs present within a sample. Bacterial communities with higher values of CMTB suggest that they are composed of a larger proportion of putative “generalists” and are likely more resistant to environmental changes. In this study, we applied two different approaches to select bacterial OTUs for the CMTB calculation and tested if the CMTB was a comparable feature among samples.

First, we restricted the accumulated bacterial community abundance for each sample regardless of the number of bacterial OTUs. Therefore, the CMTB values were compared among samples based on a consistent abundance of bacterial communities. To do this, we ranked the bacterial OTUs of a sample by their relative abundances in descending order. For the subsequent CMTB calculation, we kept the most abundant OTUs, which represented the accumulated abundances at four different levels (i.e., 0.75, 0.80, 0.85, and 0.9). Second, we randomly selected a certain number of bacterial OTUs for each sample, irrespective of their accumulated abundances. Thus, the CMTB values were compared among samples based on a consistent number of bacterial species richness. For this purpose, we randomly selected a certain number of OTUs at four different levels (i.e., 300, 500, 700, and 900) and repeated the OTUs' selection 100 times at each level. For each level, we calculated the CMTB as the average value of these 100 sets of bacterial OTUs.

### CMRA

In this study, we related the relative abundances of every pair of bacterial OTUs among all the samples to assess the differentiation of their responses along the environmental gradient. Specifically, the response asynchrony (RA_*ij*_) was calculated as the opposite value of the coefficient (*rho*) of Spearman correlation between relative abundances of OTUs *i* and *j* with a range from −1 to 1^31^. A larger value of RA_*ij*_ suggests that OTUs *i* and *j* have a greater difference in environmental optima and respond more asynchronously along the environmental gradient. In this case, OTUs *i* and *j* may be able to compensate for each one when the environment changes.

For a given sample, the CMRA was calculated as the mean of RA_*ij*_ of all pairs of bacterial OTUs weighted by the products of their relative abundances (Equation 3).

(3) $$\mathrm{CMRA}=\frac{\sum a_{i}a_{j}{\mathrm{RA}}_{\mathrm{ij}}}{\sum {a_{i}a}_{j}}$$

where *a*_*i*_ and *a*_*j*_ are the relative abundances of OTUs *i* and *j*.

Hence, a larger value of CMRA suggests that different bacterial OTUs within this community tend to respond more differentially to environmental changes and could broaden the range of environmental tolerance at the community level. Consistent with the CMTB mentioned above, the calculation of CMRA was conducted based on the bacterial OTUs that were selected using two approaches at four different levels.

### Statistical analyses

All statistical analyses were conducted in R version 3.6.1^71^ using various packages. We applied hierarchical cluster analysis to divide the 28 forest reserves into groups according to their edaphic and climatic conditions. We tested for the significant differences in environmental properties using the PERMANOVA test (*Adonis* function, Euclidean distance with permutations = 999, “vegan” package)^72^.

We conducted Pearson correlations to examine the relationships between the values of CMTB (or CMRA) that were calculated using different approaches at different levels. We related the bacterial species richness to CMTB (or CMRA) using linear and quadratic regressions. The best model was selected by identifying the model with the lowest Akaike information criteria (AIC) index. Moreover, we explored the relationships between the richness and the relative abundances of putative “generalists” (or “specialists”). For this purpose, we defined putative “generalists” and “specialists” at five quantiles (“generalists”: 0.95, 0.90, 0.85, 0.80, and 0.75; “specialists”: 0.05, 0.10, 0.15, 0.20, and 0.25) according to the distribution of TB values among bacterial OTUs (Figure S1A).

Given that communities with higher degrees of CMTB and CMRA were expected to be more resistant to environmental changes, we further assessed the potential of community‐level resistance by considering both indexes according to Equation 4.

(4) $$\mathrm{Resistance}\;\mathrm{potential}=\sqrt{{\mathrm{CMTB}}_{\mathrm{stand}}^{2}+{\text{CMRA}}_{\mathrm{stand}}^{2}}$$

where CMTB_stand_ and CMRA_stand_ are the values of a given sample standardized with a range from 0 to 1.

In addition, we identified the relative importance of the six environmental variables for this resistance potential using random forest analysis (“randomForest” package)^73^. We used seasonal dynamics of climate conditions at the annual timescale to reflect the environmental fluctuation across 28 forest reserves along the latitudinal gradient. We downloaded public climate data sets (1952–2012) across China from the National Meteorological Information Center (http://data.cma.cn/en)^61^. For each forest ecosystem, we calculated the coefficient of variation (CV) of the monthly values of each year and then calculated the mean of CV values over the 60‐year period to estimate the annual climate variation.

## AUTHOR CONTRIBUTIONS

All authors contributed to the intellectual development of this study. Jialiang Kuang conceived the research. Jialiang Kuang and Daliang Ning constructed the conceptual framework. Jialiang Kuang, Dongmei Deng, and Shun Han performed data analyses and statistics. Jialiang Kuang, Dongmei Deng, and Shun Han drafted the manuscript with help from Colin T. Bates, Wensheng Shu, and Jizhong Zhou.

## ETHICS STATEMENT

The authors acknowledge that they are scientifically and professionally involved with the interdependence of natural and technological systems. They are dedicated to the acquisition and dissemination of knowledge that advances the sciences and professions involving microbiology and ecology.

## CONFLICT OF INTERESTS

The authors declare no conflict of interests.

## Supporting information

Supporting information.

Supporting information.

Supporting information.

## Data Availability

The representative sequences of bacterial OTUs and their abundances in each sample have been deposited in figshare (https://doi.org/10.6084/m9.figshare.17711495).
