# Supplementary material for: Resistance potential of soil bacterial communities along a biodiversity gradient in forest ecosystems
Source: mLife. 2022 Nov 3;1(4):399–411. doi: 10.1002/mlf2.12042 (PMC10989803; doi:10.1002/mlf2.12042)
Supplement: Supplementary file 1 — Supporting information. [file MLF2-1-399-s001.pdf]

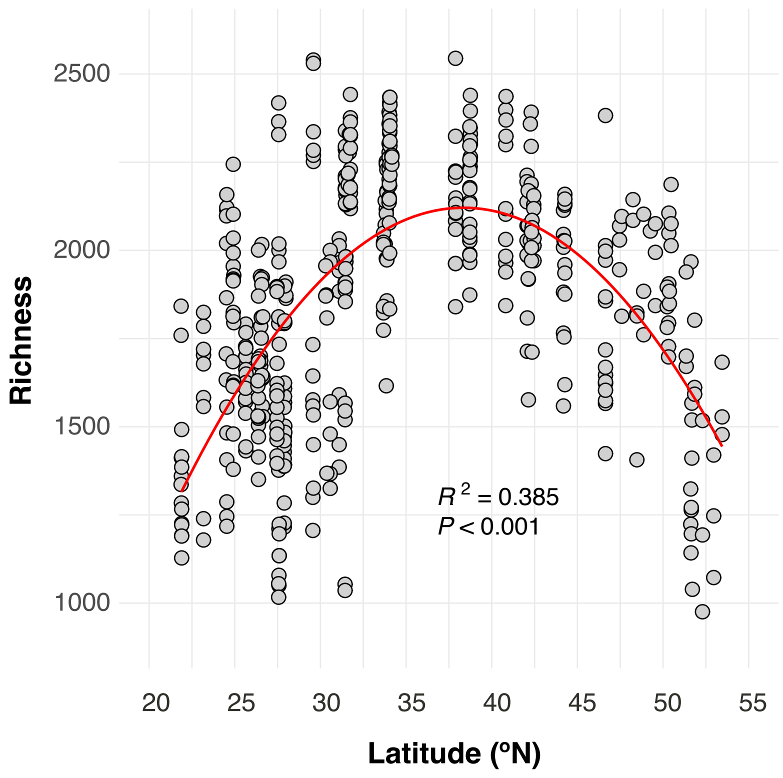

Figure S1. Relationship between bacterial species richness and latitude.

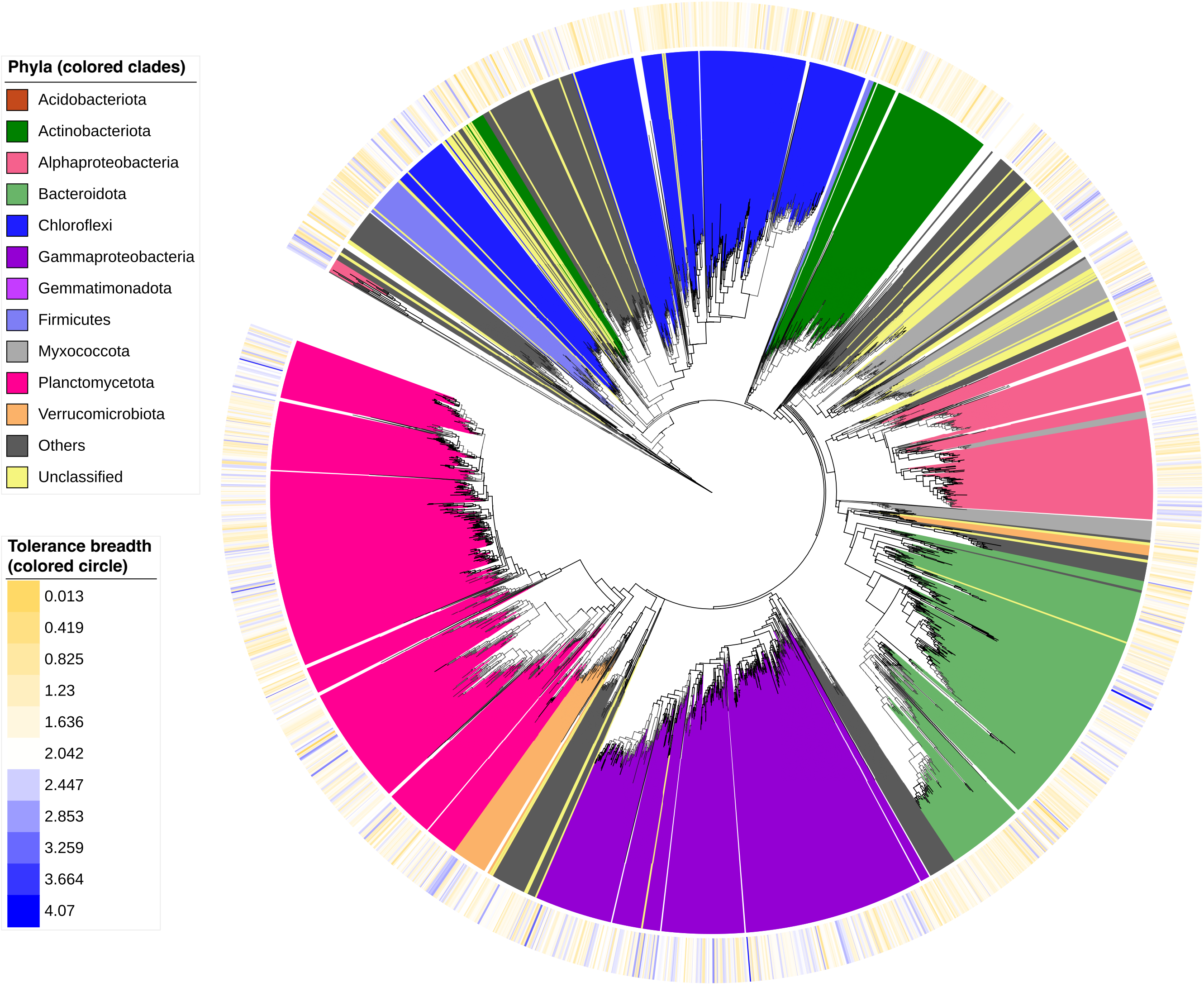

Figure S2. The circular phylogenetic tree of the bacterial species (operational taxonomic units, OTUs) and their calculated tolerance breadth (TB). The distribution of TB values was shown in Figure S3.

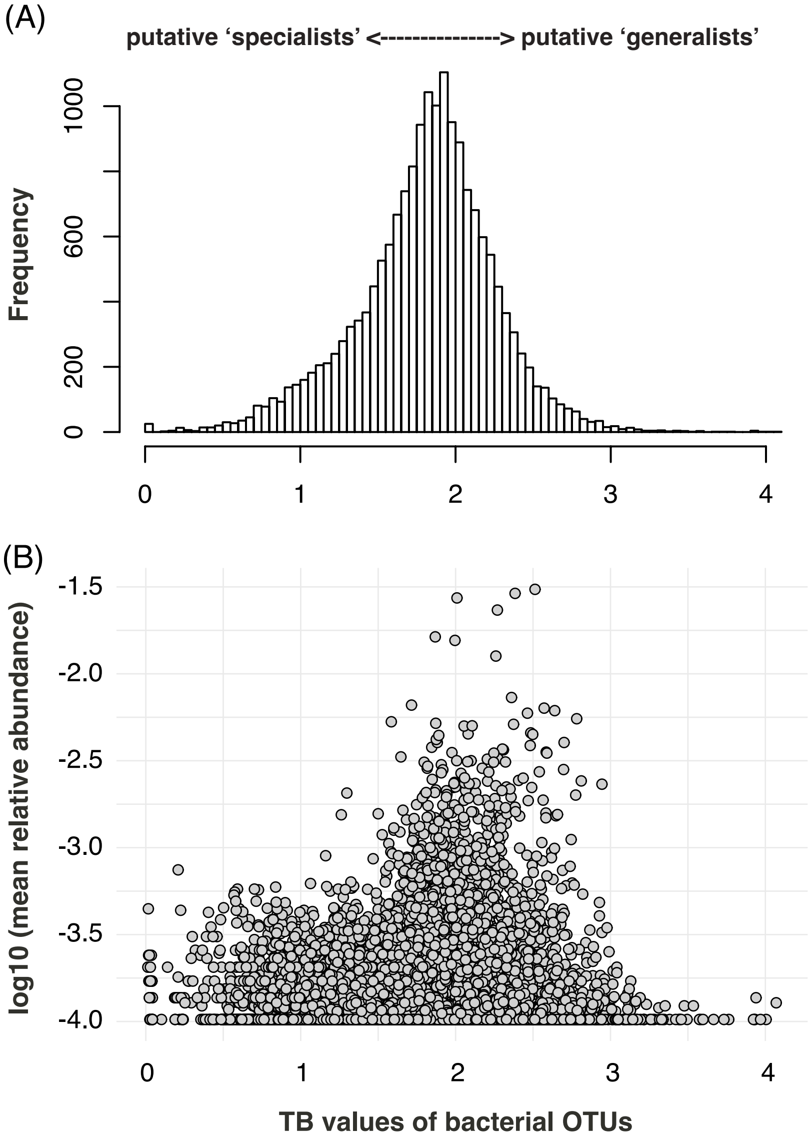

Figure S3. Distribution of TB values and the mean relative abundances among bacterial OTUs. (A) Histogram of TB values of different bacterial OTUs. (B) The mean relative abudances of different bacterial OTUs. The mean relative abudance of a given OTU was calculated based on the samples where target OTUs were detected.

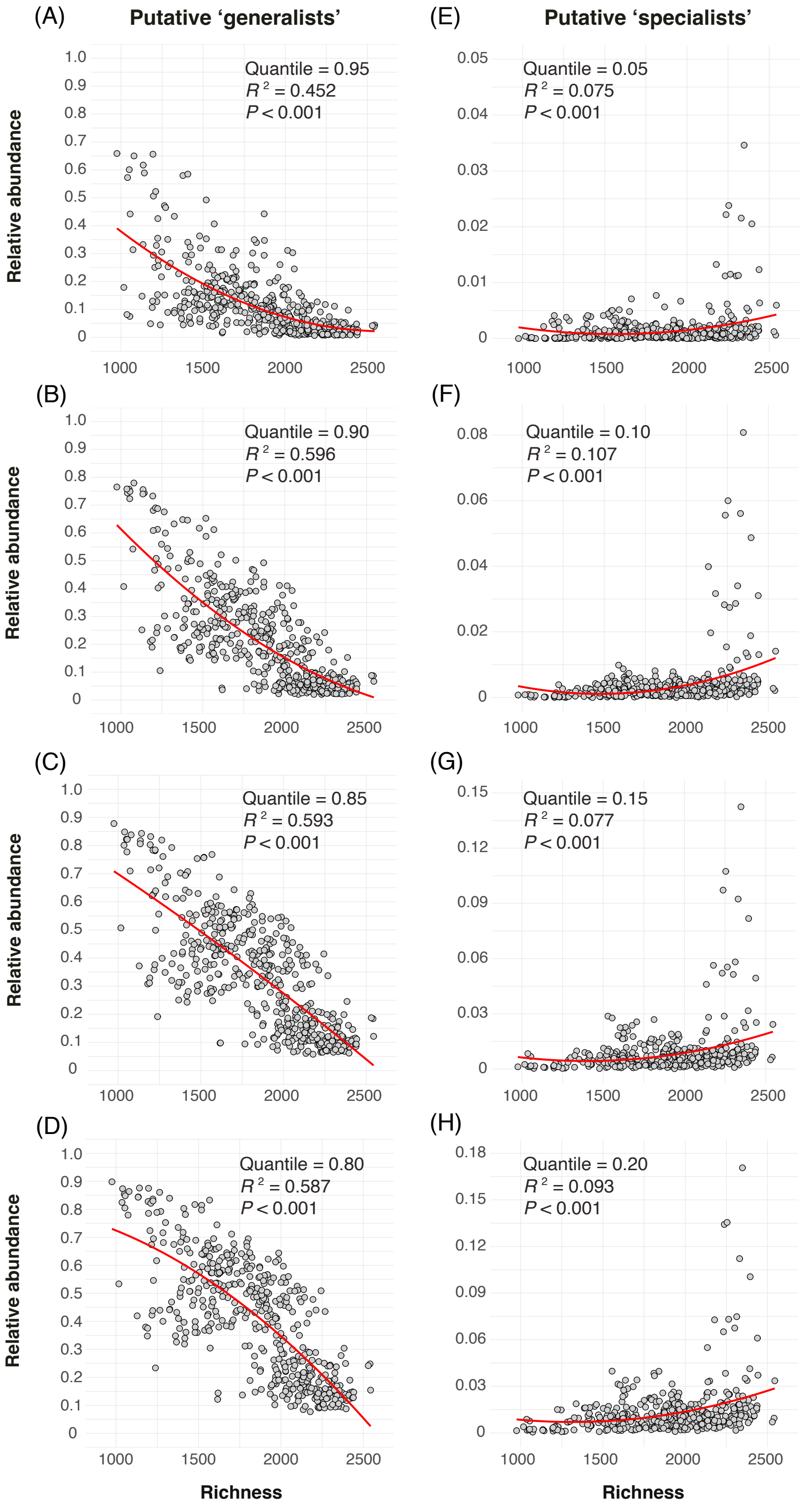

Figure S4. Relationships between bacterial species richness and the relative abundances of putative 'generalists' / 'specialists'. Putative 'generalists' (A-D) and 'specialists' (E-H) were defined based on the distribution of TB values among bacterial OTUs at five quantiles ('generalists': 0.95, 0.90, 0.85, 0.80, and 0.75; 'specialists': 0.05, 0.10, 0.15, 0.20, and 0.25). The patterns for putative 'generalists' at quantile 0.75 and putative 'specialists' at quantile 0.25 were shown in Figure 5. Quadratic regression models were selected owing to their lower values of Akaike information criteria (AIC) index than those of linear models. The distribution of TB values was shown in Figure S3.

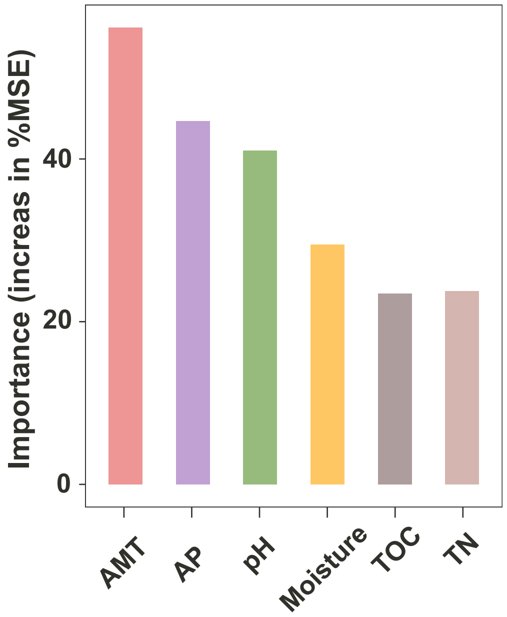

Figure S5. Predictors of resistance potential for 472 soil samples from the 28 forest reserves, identified using random forest modeling. MSE: Mean Square Error.

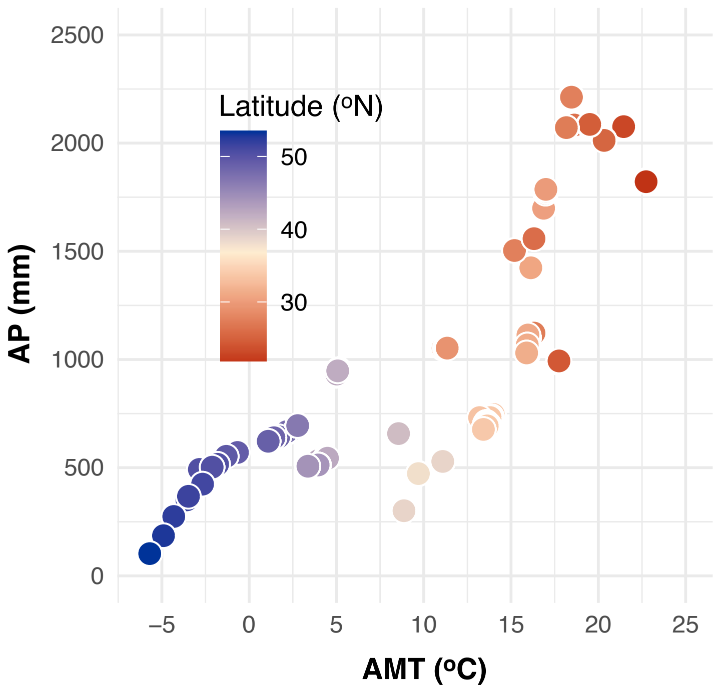

Figure S6. Annual precipitation (AP) and annual mean temperature (AMT) of different forest soil samples along latitude. Detailed information was shown in Table S1.

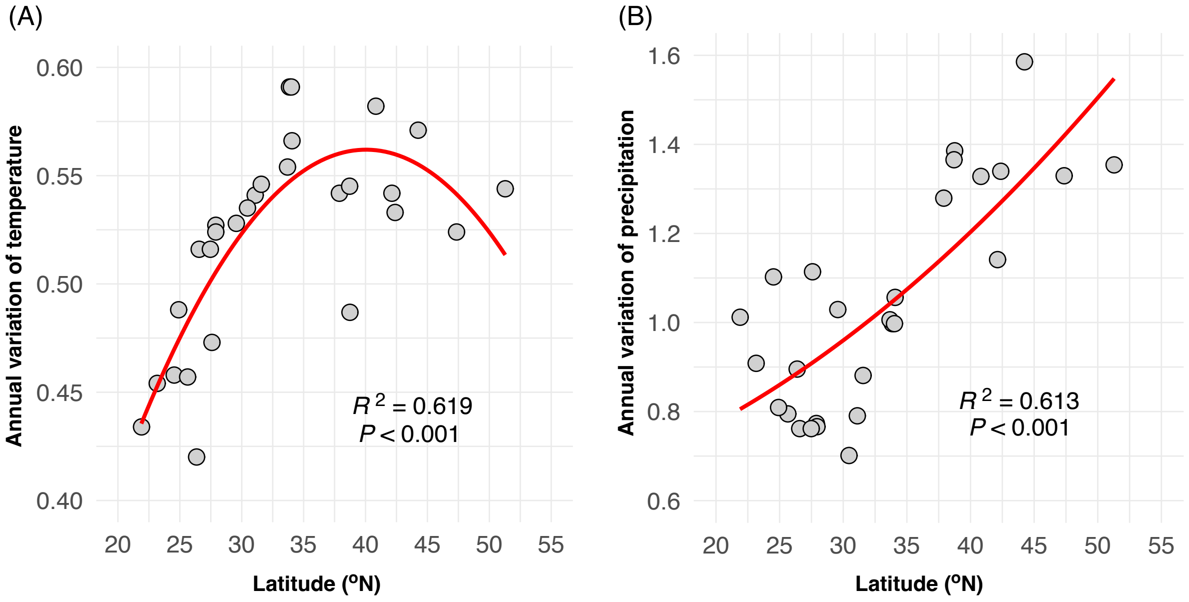

Figure S7. Relationship between latitude and annual variation of temperature and precipitation. (A) Annual variation of temperature. (B) Annual variation of precipitation. The seasonal dynamics of climate conditions at the annual timescale were used to reflect the environmental fluctuation across 28 forest reserves along the latitudinal gradient. We downloaded public climate datasets (1952-2012) across China from the National Meteorological Information Center (http://data.cma.cn/en). For each forest ecosystem, we calculated the coefficient of variation (CV) of the monthly values of each year and then calculated the mean of CV values over the 60-year period to estimate the annual climate variation. The relationships are examined using quadratic regressions.

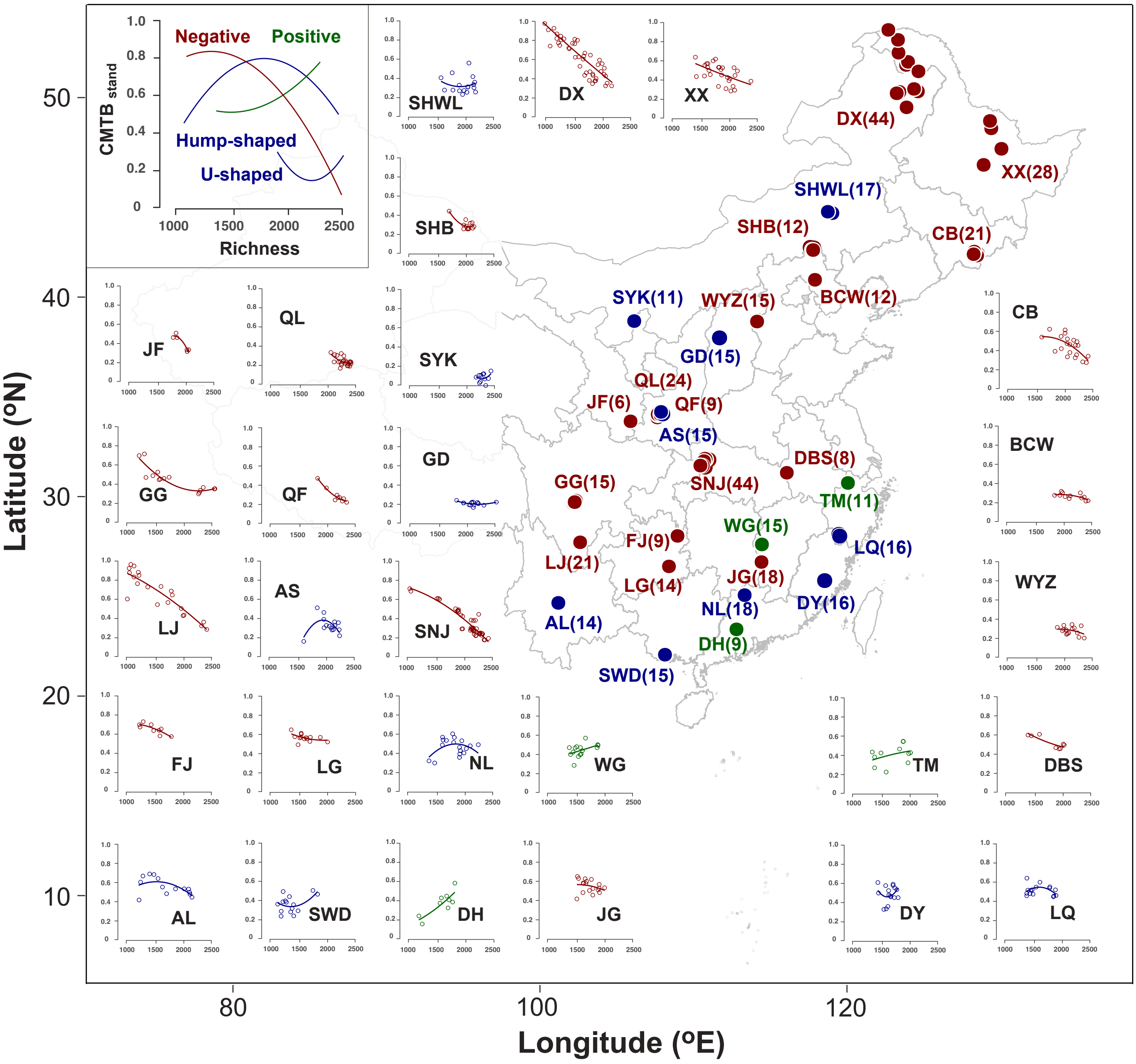

Figure S8. Relationship between community mean tolerance breadth (standardized CMTB) and bacterial species richness for soil communities within each natural forest reserve across China. The sample sizes of each forest reserve are shown in bracket. The relationships are examined using quadratic regressions. The types of relationships are differentiated by colors. In general, green plots suggest that the $CMTB_{stand}$ is positively related to the richness, while red plots represent negative relationships. In addition, the humped-shaped and U-shaped relationships are shown using blue plots.

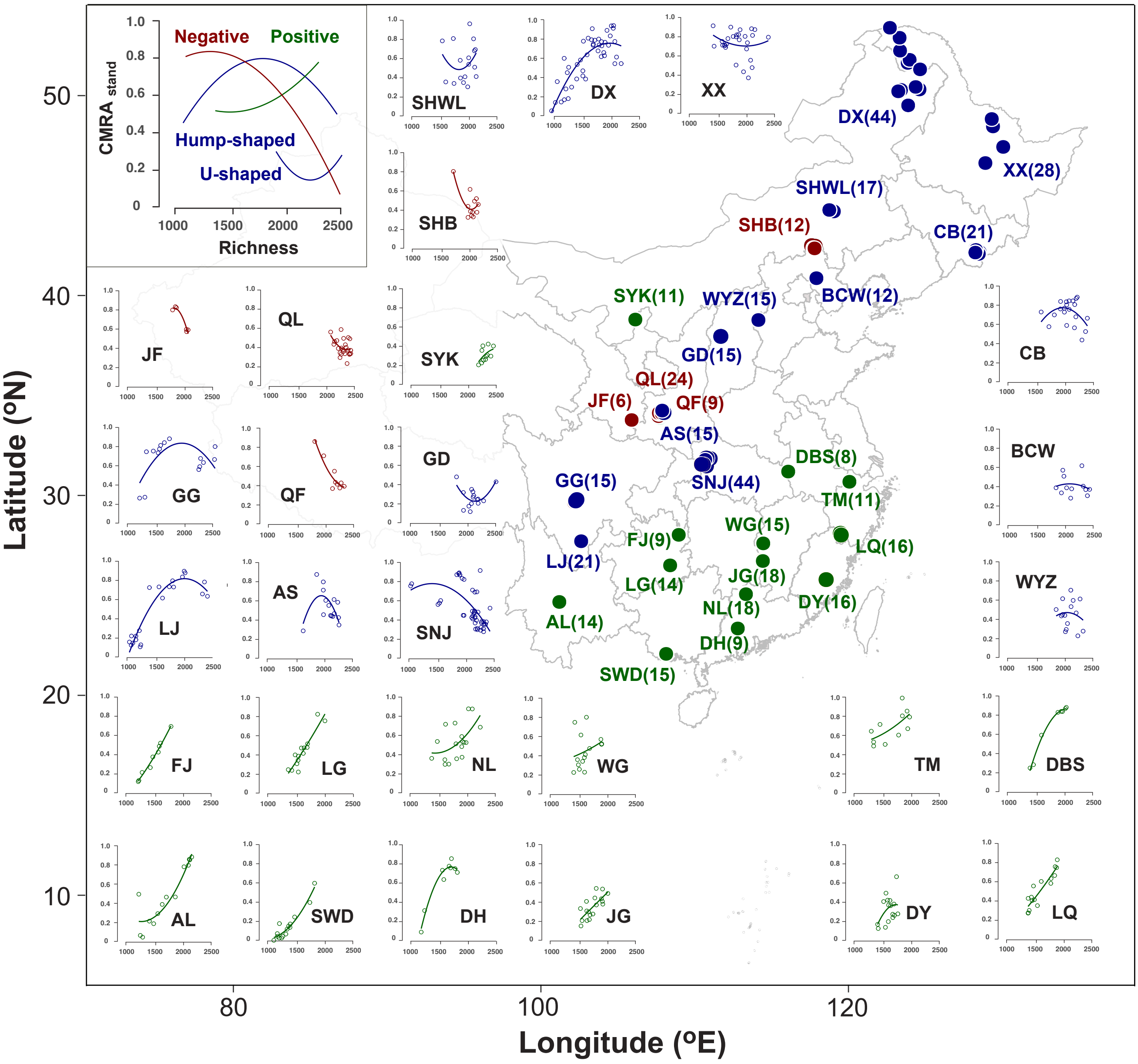

Figure S9. Relationship between community mean response asynchrony (standardized CMRA) and bacterial species richness for soil communities within each natural forest reserve across China. The sample sizes of each forest reserve are shown in bracket. The relationships are examined using quadratic regressions. The types of relationships are differentiated by colors. In general, green plots suggest that the $CMRA_{stand}$ is positively related to the richness, while red plots represent negative relationships. In addition, the humped-shaped and U-shaped relationships are shown using blue plots.

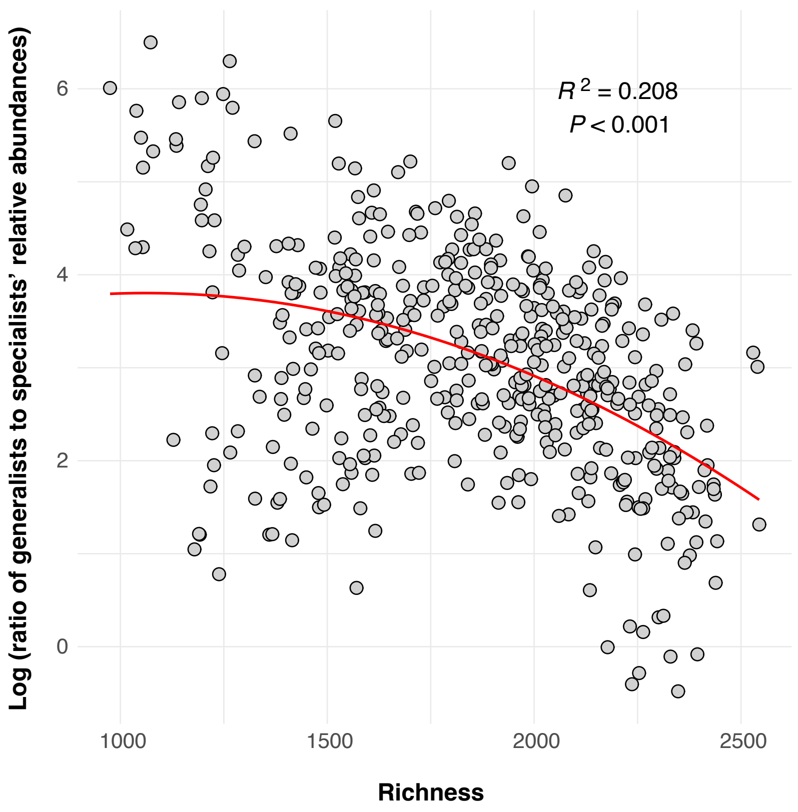

Figure S10. Relationships between bacterial species richness and the ratio of generalists to specialists' relative abundances (log-transformed). Putative 'generalists' and 'specialists' were defined based on the distribution of TB values among bacterial OTUs at quantiles of 0.75 and 0.25, respectively. Quadratic regression models were selected owing to their lower values of Akaike information criteria (AIC) index than those of linear models.

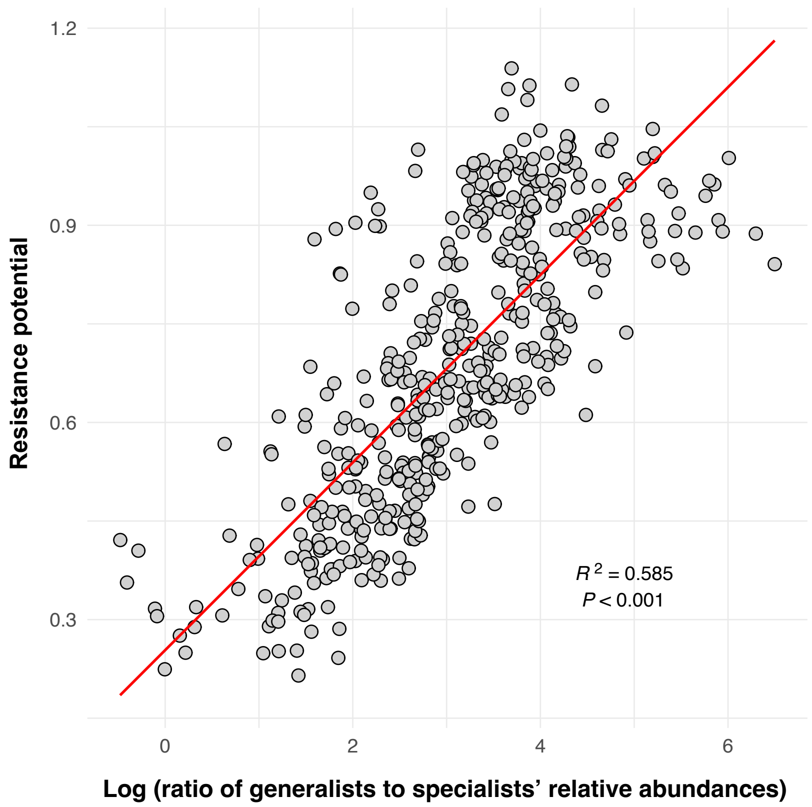

Figure S11. Relationships between resistance potential and the ratio of generalists to specialists' relative abundances (log-transformed).
