# Supplementary material for: Resistance potential of soil bacterial communities along a biodiversity gradient in forest ecosystems
Source: mLife. 2022 Nov 3;1(4):399–411. doi: 10.1002/mlf2.12042 (PMC10989803; doi:10.1002/mlf2.12042)
Supplement: Supplementary file 2 — Supporting information. [file MLF2-1-399-s003.pdf]

Table S1. Spatial location, edaphic and climatic conditions, and bacterial species richness for 472 soil samples from the 28 forest reserves in this study. TOC: total organic carbon; TN: total nitrogen; AP: annual precipitation; AMT: annual mean temperature. The grouping of forest reserves is based on the resutls of hierarchical cluster analysis (see FIGURE 2).

Samples	Forest reserve	Latitude (°N)	Longitude (°E)	pH	TOC (g kg^{-1})	TN (g kg^{-1})	Moisture (%)	AP (mm)	AMT (°C)	Richness	Group
AL1.1	Ailao Mountain	24.53	101.03	4.47	12.03	0.81	29.99	985	17.71	1246	southern region
AL1.2	Ailao Mountain	24.53	101.03	4.43	16.90	0.97	29.99	985	17.71	1406	southern region
AL1.3	Ailao Mountain	24.53	101.03	4.51	14.47	1.07	29.99	985	17.71	1483	southern region
AL2.1	Ailao Mountain	24.53	101.03	4.78	6.80	0.55	33.78	985	17.71	1218	southern region
AL2.2	Ailao Mountain	24.53	101.03	4.76	16.27	1.93	33.78	985	17.71	1556	southern region
AL2.3	Ailao Mountain	24.53	101.03	4.69	17.14	1.38	33.78	985	17.71	1287	southern region
AL3.1	Ailao Mountain	24.54	101.03	5.52	15.70	0.77	37.39	985	17.71	2114	southern region
AL3.2	Ailao Mountain	24.54	101.03	5.46	11.30	1.09	37.39	985	17.71	2122	southern region
AL3.3	Ailao Mountain	24.54	101.03	5.27	16.01	0.94	37.39	985	17.71	2158	southern region
AL4.1	Ailao Mountain	24.51	101.01	5.10	8.60	0.80	25.79	989	17.73	1866	southern region
AL4.2	Ailao Mountain	24.51	101.01	5.05	12.19	0.82	25.79	989	17.73	2098	southern region
AL4.3	Ailao Mountain	24.51	101.01	5.09	8.95	0.41	25.79	989	17.73	2020	southern region
AL5.1	Ailao Mountain	24.50	100.99	4.92	7.07	0.32	22.92	991	17.74	1633	southern region
AL5.3	Ailao Mountain	24.50	100.99	4.91	8.22	0.38	22.92	991	17.74	1707	southern region
AS1.1	Aoshan Mountain	33.86	107.47	6.13	15.23	0.76	57.49	731	13.86	1977	northern region
AS1.2	Aoshan Mountain	33.86	107.47	6.01	12.92	0.80	57.49	731	13.86	2117	northern region
AS1.3	Aoshan Mountain	33.86	107.47	6.02	11.66	0.67	57.49	731	13.86	2104	northern region
AS2.1	Aoshan Mountain	33.79	107.50	5.92	5.51	0.55	39.95	744	13.99	2072	northern region
AS2.2	Aoshan Mountain	33.79	107.50	6.14	8.84	0.34	39.95	744	13.99	2141	northern region
AS2.3	Aoshan Mountain	33.79	107.50	6.33	8.31	0.67	39.95	744	13.99	2018	northern region
AS3.1	Aoshan Mountain	33.85	107.49	6.71	15.77	0.42	31.46	733	13.88	2252	northern region
AS3.2	Aoshan Mountain	33.85	107.49	6.17	4.41	0.35	31.46	733	13.88	1616	northern region
AS3.3	Aoshan Mountain	33.85	107.49	6.54	6.46	0.19	31.46	733	13.88	2244	northern region
AS4.1	Aoshan Mountain	33.88	107.44	5.94	17.98	0.50	56.45	728	13.82	2168	northern region
AS4.2	Aoshan Mountain	33.88	107.44	6.34	14.74	0.87	56.45	728	13.82	2164	northern region
AS4.3	Aoshan Mountain	33.88	107.44	6.45	26.30	0.61	56.45	728	13.82	2229	northern region
AS5.1	Aoshan Mountain	33.88	107.41	5.82	17.96	0.47	59.76	728	13.80	1974	northern region
AS5.2	Aoshan Mountain	33.88	107.41	5.46	7.21	0.50	59.76	728	13.80	1973	northern region
AS5.3	Aoshan Mountain	33.88	107.41	5.24	14.26	0.19	59.76	728	13.80	1857	northern region
BCW1.1	Baicaowa	40.82	117.61	5.83	9.74	1.01	27.29	656	8.60	1964	northern region
BCW1.2	Baicaowa	40.82	117.61	5.64	10.55	0.92	27.29	656	8.60	1983	northern region
BCW1.3	Baicaowa	40.82	117.61	5.69	8.91	0.78	27.29	656	8.60	1843	northern region

BCW2.1	Baicaowa	40.82	117.61	5.66	9.59	0.72	20.25	655	8.58	1939	northern region
BCW2.2	Baicaowa	40.82	117.61	5.59	12.84	1.05	20.25	655	8.58	2103	northern region
BCW2.3	Baicaowa	40.82	117.61	5.70	7.82	0.57	20.25	655	8.58	2119	northern region
BCW3.1	Baicaowa	40.83	117.61	5.92	12.04	0.88	26.39	655	8.56	2300	northern region
BCW3.2	Baicaowa	40.83	117.61	5.81	10.73	0.83	26.39	655	8.56	2398	northern region
BCW3.3	Baicaowa	40.83	117.61	5.86	7.85	0.57	26.39	655	8.56	2031	northern region
BCW5.1	Baicaowa	40.83	117.60	6.08	4.16	0.21	20.86	654	8.55	2437	northern region
BCW5.2	Baicaowa	40.83	117.60	5.87	10.34	0.84	20.86	654	8.55	2370	northern region
BCW5.3	Baicaowa	40.83	117.60	5.92	4.39	0.43	20.86	654	8.55	2324	northern region
CB2.1	Changbai Mountain	42.14	128.13	6.15	4.40	0.17	51.42	941	5.09	1918	northern region
CB2.2	Changbai Mountain	42.14	128.13	6.38	3.76	0.24	51.42	941	5.09	1577	northern region
CB2.3	Changbai Mountain	42.14	128.13	6.20	4.40	0.31	51.42	941	5.09	2021	northern region
CB3.1	Changbai Mountain	42.08	128.07	6.31	11.17	0.88	57.18	948	5.12	1985	northern region
CB3.2	Changbai Mountain	42.08	128.07	5.80	6.35	0.67	57.18	948	5.12	1714	northern region
CB3.3	Changbai Mountain	42.08	128.07	6.05	6.71	0.85	57.18	948	5.12	1809	northern region
CB4.1	Changbai Mountain	42.06	128.07	6.02	7.22	0.50	55.39	950	5.13	2138	northern region
CB4.2	Changbai Mountain	42.06	128.07	6.20	7.53	0.43	55.39	950	5.13	2028	northern region
CB4.3	Changbai Mountain	42.06	128.07	6.27	7.93	0.36	55.39	950	5.13	1987	northern region
CB5.1	Changbai Mountain	42.06	128.07	5.59	7.36	0.45	51.09	950	5.13	2070	northern region
CB5.2	Changbai Mountain	42.06	128.07	6.03	6.60	0.35	51.09	950	5.13	2213	northern region
CB5.3	Changbai Mountain	42.06	128.07	5.83	8.77	0.40	51.09	950	5.13	2195	northern region
CB6.1	Changbai Mountain	42.30	127.83	5.58	9.21	0.85	60.32	924	4.98	2189	northern region
CB6.2	Changbai Mountain	42.30	127.83	5.78	13.90	0.85	60.32	924	4.98	2392	northern region
CB6.3	Changbai Mountain	42.30	127.83	5.65	14.01	1.27	60.32	924	4.98	2295	northern region
CB7.1	Changbai Mountain	42.26	127.89	4.96	6.49	0.82	46.30	929	5.01	2028	northern region
CB7.2	Changbai Mountain	42.26	127.89	5.01	10.41	1.00	46.30	929	5.01	2164	northern region
CB7.3	Changbai Mountain	42.26	127.89	5.18	10.35	0.65	46.30	929	5.01	2359	northern region
CB8.1	Changbai Mountain	42.12	127.85	5.30	7.56	0.59	56.42	944	5.07	2071	northern region
CB8.2	Changbai Mountain	42.12	127.85	5.36	8.59	1.23	56.42	944	5.07	2170	northern region
CB8.3	Changbai Mountain	42.12	127.85	5.44	6.70	0.69	56.42	944	5.07	1920	northern region
DBS1.3	Dabie Mountain	31.10	115.77	5.15	3.20	0.24	51.38	1415	16.13	1591	southern region
DBS2.1	Dabie Mountain	31.09	115.77	5.13	8.17	0.65	37.60	1419	16.13	1449	southern region
DBS2.2	Dabie Mountain	31.09	115.77	5.12	8.17	0.65	37.60	1419	16.13	1386	southern region
DBS3.1	Dabie Mountain	31.09	115.78	4.64	6.77	0.43	26.58	1420	16.13	1942	southern region
DBS3.2	Dabie Mountain	31.09	115.78	4.66	6.77	0.41	26.58	1420	16.13	1884	southern region
DBS3.3	Dabie Mountain	31.09	115.78	4.64	6.77	0.42	26.58	1420	16.13	1965	southern region
DBS4.1	Dabie Mountain	31.09	115.81	4.93	14.24	0.85	15.24	1421	16.13	2033	southern region
DBS4.2	Dabie Mountain	31.09	115.81	4.89	14.24	0.63	15.24	1421	16.13	2014	southern region

DH1.1	Dinghu Mountain	23.17	112.54	4.57	1.81	0.16	23.92	2073	21.46	1824	southern region
DH1.2	Dinghu Mountain	23.17	112.54	4.71	1.31	0.12	23.92	2073	21.46	1239	southern region
DH1.3	Dinghu Mountain	23.17	112.54	4.47	1.49	0.11	23.92	2073	21.46	1179	southern region
DH2.1	Dinghu Mountain	23.17	112.53	4.37	4.45	0.22	18.73	2073	21.46	1583	southern region
DH2.2	Dinghu Mountain	23.17	112.53	4.28	3.56	0.29	18.73	2073	21.46	1784	southern region
DH2.3	Dinghu Mountain	23.17	112.53	4.23	4.15	0.21	18.73	2073	21.46	1703	southern region
DH3.1	Dinghu Mountain	23.18	112.52	4.01	4.81	0.26	22.01	2073	21.45	1678	southern region
DH3.2	Dinghu Mountain	23.18	112.52	4.08	3.44	0.25	22.01	2073	21.45	1719	southern region
DH3.3	Dinghu Mountain	23.18	112.52	4.07	4.19	0.19	22.01	2073	21.45	1558	southern region
DX1.2	Daxing'anling	49.25	123.80	6.72	15.84	0.92	21.28	567	-0.67	2055	northern region
DX10.1	Daxing'anling	51.62	123.53	4.49	27.23	0.77	48.93	372	-3.48	1324	northern region
DX10.3	Daxing'anling	51.62	123.53	4.48	21.59	0.42	48.93	372	-3.48	1223	northern region
DX11.2	Daxing'anling	51.63	123.52	4.03	13.96	0.58	12.77	371	-3.49	1967	northern region
DX11.3	Daxing'anling	51.63	123.52	3.67	14.11	0.14	12.77	371	-3.49	1142	northern region
DX12.1	Daxing'anling	51.63	123.52	4.49	4.75	0.25	54.32	371	-3.49	1264	northern region
DX12.2	Daxing'anling	51.63	123.52	5.01	17.46	0.11	54.32	371	-3.49	1197	northern region
DX12.3	Daxing'anling	51.63	123.52	4.90	7.17	0.23	54.32	371	-3.49	1271	northern region
DX13.1	Daxing'anling	51.68	123.49	4.82	19.67	0.69	49.14	364	-3.55	1411	northern region
DX13.2	Daxing'anling	51.68	123.49	4.85	25.63	0.15	49.14	364	-3.55	1567	northern region
DX13.3	Daxing'anling	51.68	123.49	4.93	7.21	0.54	49.14	364	-3.55	1519	northern region
DX14.1	Daxing'anling	51.84	123.61	5.04	14.98	0.70	35.86	348	-3.57	1802	northern region
DX14.2	Daxing'anling	51.84	123.61	5.22	15.62	0.65	35.86	348	-3.57	1612	northern region
DX14.3	Daxing'anling	51.84	123.61	4.88	20.40	0.32	35.86	348	-3.57	1592	northern region
DX15.1	Daxing'anling	52.30	122.99	4.14	13.84	0.27	68.14	272	-4.32	975	northern region
DX15.2	Daxing'anling	52.30	122.99	4.23	11.50	0.55	68.14	272	-4.32	1194	northern region
DX15.3	Daxing'anling	52.30	122.99	4.30	18.85	0.44	68.14	272	-4.32	1518	northern region
DX16.1	Daxing'anling	52.95	122.96	4.49	15.69	0.20	38.49	183	-4.90	1248	northern region
DX16.2	Daxing'anling	52.95	122.96	4.86	10.72	0.39	38.49	183	-4.90	1073	northern region
DX16.3	Daxing'anling	52.95	122.96	4.47	14.04	0.41	38.49	183	-4.90	1420	northern region
DX17.1	Daxing'anling	53.45	122.34	4.84	12.38	0.71	24.96	100	-5.69	1528	northern region
DX17.2	Daxing'anling	53.45	122.34	4.67	29.44	0.66	24.96	100	-5.69	1478	northern region
DX17.3	Daxing'anling	53.45	122.34	4.94	34.79	0.26	24.96	100	-5.69	1683	northern region
DX2.1	Daxing'anling	49.54	123.51	6.53	8.52	1.28	35.04	549	-1.31	1995	northern region
DX2.2	Daxing'anling	49.54	123.51	6.42	22.79	1.35	35.04	549	-1.31	2076	northern region
DX2.3	Daxing'anling	49.54	123.51	6.72	19.97	0.87	35.04	549	-1.31	1844	northern region
DX3.1	Daxing'anling	50.34	124.24	6.30	13.14	1.48	34.62	513	-1.80	1905	northern region
DX3.2	Daxing'anling	50.34	124.24	6.91	18.79	1.29	34.62	513	-1.80	2106	northern region
DX3.3	Daxing'anling	50.34	124.24	6.69	12.34	0.66	34.62	513	-1.80	2048	northern region

DX4.1	Daxing'anling	50.32	123.05	6.58	10.04	0.37	32.19	493	-2.79	1794	northern region
DX4.2	Daxing'anling	50.32	123.05	6.47	7.83	0.41	32.19	493	-2.79	1727	northern region
DX4.3	Daxing'anling	50.32	123.05	6.44	6.91	0.46	32.19	493	-2.79	1698	northern region
DX5.1	Daxing'anling	50.25	122.88	6.71	8.66	0.67	19.10	495	-2.86	1782	northern region
DX5.2	Daxing'anling	50.25	122.88	6.37	12.63	1.01	19.10	495	-2.86	1896	northern region
DX5.3	Daxing'anling	50.25	122.88	6.55	7.06	0.53	19.10	495	-2.86	1840	northern region
DX6.1	Daxing'anling	50.36	123.03	6.37	10.24	0.79	14.44	489	-2.85	1849	northern region
DX6.3	Daxing'anling	50.36	123.03	6.17	8.92	0.61	14.44	489	-2.85	1884	northern region
DX7.1	Daxing'anling	50.47	124.00	6.22	18.58	1.40	20.59	499	-2.12	2187	northern region
DX7.2	Daxing'anling	50.47	124.00	6.24	24.05	1.30	20.59	499	-2.12	2014	northern region
DX7.3	Daxing'anling	50.47	124.00	6.45	14.15	0.89	20.59	499	-2.12	2076	northern region
DX8.1	Daxing'anling	51.36	124.28	6.11	19.84	1.10	44.03	421	-2.66	1671	northern region
DX8.2	Daxing'anling	51.36	124.28	6.18	24.26	1.25	44.03	421	-2.66	1939	northern region
DX8.3	Daxing'anling	51.36	124.28	6.05	20.07	0.93	44.03	421	-2.66	1701	northern region
DX9.1	Daxing'anling	51.70	123.62	5.62	12.63	0.84	29.22	365	-3.47	1039	northern region
DY1.1	Daiyun Mountain	25.64	118.22	4.45	15.76	0.71	41.27	2010	20.33	1571	southern region
DY1.2	Daiyun Mountain	25.64	118.22	4.66	11.72	0.62	41.27	2010	20.33	1728	southern region
DY1.3	Daiyun Mountain	25.64	118.22	4.80	8.32	0.43	41.27	2010	20.33	1621	southern region
DY2.1	Daiyun Mountain	25.65	118.22	4.60	19.46	1.33	45.30	2011	20.33	1701	southern region
DY2.2	Daiyun Mountain	25.65	118.22	4.76	6.75	1.32	45.30	2011	20.33	1699	southern region
DY2.3	Daiyun Mountain	25.65	118.22	4.86	6.88	0.59	45.30	2011	20.33	1791	southern region
DY3.1	Daiyun Mountain	25.65	118.22	4.62	26.51	0.87	45.10	2012	20.33	1432	southern region
DY3.2	Daiyun Mountain	25.65	118.22	4.71	7.28	0.38	45.10	2012	20.33	1443	southern region
DY3.3	Daiyun Mountain	25.65	118.22	4.65	7.59	0.71	45.10	2012	20.33	1767	southern region
DY4.1	Daiyun Mountain	25.65	118.23	4.66	8.47	0.39	35.50	2012	20.33	1662	southern region
DY4.2	Daiyun Mountain	25.65	118.23	4.44	6.88	0.55	35.50	2012	20.33	1722	southern region
DY5.1	Daiyun Mountain	25.65	118.22	5.01	11.70	0.52	38.22	2012	20.32	1650	southern region
DY5.2	Daiyun Mountain	25.65	118.22	4.85	13.50	0.38	38.22	2012	20.32	1626	southern region
DY6.1	Daiyun Mountain	25.64	118.22	5.20	7.27	0.66	35.32	2010	20.33	1580	southern region
DY6.2	Daiyun Mountain	25.64	118.22	5.39	3.05	0.24	35.32	2010	20.33	1538	southern region
DY6.3	Daiyun Mountain	25.64	118.22	5.29	4.90	0.34	35.32	2010	20.33	1609	southern region
FJ2.1	Fanjing Mountain	27.91	108.70	4.03	12.67	0.07	66.33	1500	15.18	1569	southern region
FJ2.2	Fanjing Mountain	27.91	108.70	3.89	14.96	1.40	66.33	1500	15.18	1608	southern region
FJ2.3	Fanjing Mountain	27.91	108.70	4.54	10.44	1.17	66.33	1500	15.18	1586	southern region
FJ3.1	Fanjing Mountain	27.90	108.71	3.90	18.45	1.31	59.62	1501	15.19	1429	southern region
FJ3.2	Fanjing Mountain	27.90	108.71	3.79	18.07	1.07	59.62	1501	15.19	1472	southern region
FJ3.3	Fanjing Mountain	27.90	108.71	3.79	13.24	0.79	59.62	1501	15.19	1215	southern region
FJ4.1	Fanjing Mountain	27.90	108.72	3.89	21.02	1.44	39.85	1501	15.19	1792	southern region

FJ4.2	Fanjing Mountain	27.90	108.72	4.03	21.08	2.50	39.85	1501	15.19	1227	southern region
FJ4.3	Fanjing Mountain	27.90	108.72	3.84	17.48	1.36	39.85	1501	15.19	1284	southern region
GD1.1	Guandi Mountain	37.89	111.44	6.16	6.47	0.57	37.92	469	9.71	2323	northern region
GD1.2	Guandi Mountain	37.89	111.44	6.34	6.93	0.48	37.92	469	9.71	2544	northern region
GD1.3	Guandi Mountain	37.89	111.44	6.27	6.66	0.48	37.92	469	9.71	2225	northern region
GD2.1	Guandi Mountain	37.89	111.44	5.60	6.53	0.42	43.51	469	9.71	1963	northern region
GD2.2	Guandi Mountain	37.89	111.44	6.38	12.18	0.53	43.51	469	9.71	2083	northern region
GD2.3	Guandi Mountain	37.89	111.44	6.46	5.23	0.29	43.51	469	9.71	2060	northern region
GD3.1	Guandi Mountain	37.89	111.43	5.91	8.17	0.54	56.00	469	9.70	2207	northern region
GD3.2	Guandi Mountain	37.89	111.43	6.30	5.60	0.56	56.00	469	9.70	1840	northern region
GD3.3	Guandi Mountain	37.89	111.43	6.58	5.16	0.50	56.00	469	9.70	1962	northern region
GD4.1	Guandi Mountain	37.89	111.44	6.74	4.53	0.39	43.02	469	9.70	2222	northern region
GD4.2	Guandi Mountain	37.89	111.44	6.82	4.32	0.42	43.02	469	9.70	2135	northern region
GD4.3	Guandi Mountain	37.89	111.44	6.47	8.08	0.71	43.02	469	9.70	2148	northern region
GD5.1	Guandi Mountain	37.90	111.43	6.60	6.69	0.54	43.90	469	9.69	2105	northern region
GD5.2	Guandi Mountain	37.90	111.43	6.47	8.67	0.71	43.90	469	9.69	2131	northern region
GD5.3	Guandi Mountain	37.90	111.43	6.81	5.64	0.44	43.90	469	9.69	2108	northern region
GG1.1	Gongga Mountain	29.54	101.96	6.66	2.53	0.04	32.87	1049	11.09	1577	southern region
GG1.2	Gongga Mountain	29.54	101.96	6.66	2.53	0.06	32.87	1049	11.09	1644	southern region
GG1.3	Gongga Mountain	29.54	101.96	6.66	2.53	0.05	32.87	1049	11.09	1733	southern region
GG2.1	Gongga Mountain	29.55	101.96	5.57	7.61	0.10	26.71	1049	11.10	1206	southern region
GG2.2	Gongga Mountain	29.55	101.96	5.57	7.61	0.14	26.71	1049	11.10	1559	southern region
GG2.3	Gongga Mountain	29.55	101.96	5.57	7.61	0.14	26.71	1049	11.10	1300	southern region
GG3.1	Gongga Mountain	29.59	102.03	4.05	5.71	0.05	25.40	1049	11.24	1534	southern region
GG3.2	Gongga Mountain	29.59	102.03	4.05	5.71	0.08	25.40	1049	11.24	1449	southern region
GG3.3	Gongga Mountain	29.59	102.03	4.05	5.71	0.09	25.40	1049	11.24	1326	southern region
GG4.1	Gongga Mountain	29.60	102.04	3.80	8.01	0.12	22.82	1049	11.29	2336	southern region
GG4.2	Gongga Mountain	29.60	102.04	3.80	8.01	0.15	22.82	1049	11.29	2540	southern region
GG4.3	Gongga Mountain	29.60	102.04	3.80	8.01	0.14	22.82	1049	11.29	2530	southern region
GG5.1	Gongga Mountain	29.60	102.07	3.73	10.21	0.09	18.65	1050	11.35	2252	southern region
GG5.2	Gongga Mountain	29.60	102.07	3.73	10.21	0.07	18.65	1050	11.35	2270	southern region
GG5.3	Gongga Mountain	29.60	102.07	3.73	10.21	0.10	18.65	1050	11.35	2284	southern region
JF1.1	Jifeng Mountain	33.69	105.68	5.12	3.98	0.28	56.68	728	13.19	1773	northern region
JF1.2	Jifeng Mountain	33.69	105.68	4.95	13.58	0.72	56.68	728	13.19	1823	northern region
JF1.3	Jifeng Mountain	33.69	105.68	5.08	5.65	0.40	56.68	728	13.19	1841	northern region
JF2.1	Jifeng Mountain	33.68	105.68	4.88	7.79	0.32	50.39	728	13.19	2049	northern region
JF2.2	Jifeng Mountain	33.68	105.68	4.94	13.23	0.46	50.39	728	13.19	2023	northern region
JF2.3	Jifeng Mountain	33.68	105.68	5.14	4.89	0.31	50.39	728	13.19	2017	northern region

JG1.1	Jinggang Mountain	26.50	114.16	4.20	15.07	1.04	67.52	2084	18.67	1539	southern region
JG1.2	Jinggang Mountain	26.50	114.16	4.15	13.36	0.58	67.52	2084	18.67	1700	southern region
JG1.3	Jinggang Mountain	26.50	114.16	4.27	7.07	0.30	67.52	2084	18.67	1926	southern region
JG2.1	Jinggang Mountain	26.51	114.17	4.00	24.10	0.50	65.72	2084	18.67	1674	southern region
JG2.2	Jinggang Mountain	26.51	114.17	4.02	28.87	0.62	65.72	2084	18.67	1785	southern region
JG2.3	Jinggang Mountain	26.51	114.17	4.04	11.39	0.32	65.72	2084	18.67	1688	southern region
JG3.1	Jinggang Mountain	26.60	114.13	4.83	15.61	0.42	48.43	2080	18.60	1559	southern region
JG3.2	Jinggang Mountain	26.60	114.13	4.74	7.75	0.43	48.43	2080	18.60	1923	southern region
JG3.3	Jinggang Mountain	26.60	114.13	4.92	4.46	0.31	48.43	2080	18.60	1637	southern region
JG4.1	Jinggang Mountain	26.55	114.12	4.99	5.96	0.50	49.67	2081	18.63	1810	southern region
JG4.2	Jinggang Mountain	26.55	114.12	5.00	4.15	0.29	49.67	2081	18.63	1919	southern region
JG4.3	Jinggang Mountain	26.55	114.12	4.74	4.40	0.34	49.67	2081	18.63	1751	southern region
JG5.1	Jinggang Mountain	26.63	114.11	4.64	5.58	0.47	33.93	2079	18.58	1642	southern region
JG5.2	Jinggang Mountain	26.63	114.11	4.59	4.28	0.29	33.93	2079	18.58	1812	southern region
JG5.3	Jinggang Mountain	26.63	114.11	4.79	2.97	0.18	33.93	2079	18.58	1529	southern region
JG6.1	Jinggang Mountain	26.58	114.14	4.20	5.72	0.43	34.80	2081	18.62	1903	southern region
JG6.2	Jinggang Mountain	26.58	114.14	4.02	6.24	2.75	34.80	2081	18.62	2017	southern region
JG6.3	Jinggang Mountain	26.58	114.14	3.98	12.67	0.31	34.80	2081	18.62	1646	southern region
LG1.1	Leigong Mountain	26.38	108.21	4.73	3.00	0.33	41.26	1559	16.31	1505	southern region
LG1.2	Leigong Mountain	26.38	108.21	4.14	10.90	0.08	41.26	1559	16.31	1582	southern region
LG1.3	Leigong Mountain	26.38	108.21	4.52	10.10	0.09	41.26	1559	16.31	1692	southern region
LG2.1	Leigong Mountain	26.38	108.20	4.31	9.22	0.06	21.96	1558	16.31	1472	southern region
LG2.2	Leigong Mountain	26.38	108.20	5.03	12.96	0.08	21.96	1558	16.31	1684	southern region
LG3.1	Leigong Mountain	26.38	108.20	4.37	8.32	0.06	25.45	1558	16.31	1524	southern region
LG3.2	Leigong Mountain	26.38	108.20	4.40	8.91	0.08	25.45	1558	16.31	1351	southern region
LG3.3	Leigong Mountain	26.38	108.20	4.34	8.39	0.10	25.45	1558	16.31	1414	southern region
LG4.1	Leigong Mountain	26.37	108.18	4.45	10.46	0.06	32.05	1556	16.31	2001	southern region
LG4.2	Leigong Mountain	26.37	108.18	4.68	8.75	0.13	32.05	1556	16.31	1871	southern region
LG4.3	Leigong Mountain	26.37	108.18	4.46	11.62	0.09	32.05	1556	16.31	1529	southern region
LG5.1	Leigong Mountain	26.36	108.16	4.68	9.22	0.06	15.81	1555	16.31	1616	southern region
LG5.2	Leigong Mountain	26.36	108.16	4.82	10.06	0.06	15.81	1555	16.31	1631	southern region
LG5.3	Leigong Mountain	26.36	108.16	4.42	13.89	0.17	15.81	1555	16.31	1531	southern region
LJ1.1	Luoji Mountain	27.58	102.38	3.97	25.97	1.11	52.91	1118	16.29	1050	southern region
LJ1.2	Luoji Mountain	27.58	102.38	4.20	36.65	1.36	52.91	1118	16.29	1079	southern region
LJ1.3	Luoji Mountain	27.58	102.38	4.62	32.92	2.08	52.91	1118	16.29	1211	southern region
LJ2.1	Luoji Mountain	27.58	102.38	5.17	39.87	1.32	55.25	1119	16.30	1135	southern region
LJ2.2	Luoji Mountain	27.58	102.38	5.19	32.63	1.57	55.25	1119	16.30	1224	southern region
LJ2.3	Luoji Mountain	27.58	102.38	4.68	34.35	1.13	55.25	1119	16.30	1134	southern region

LJ3.1	Luoji Mountain	27.58	102.39	4.57	12.18	0.76	39.44	1119	16.30	1549	southern region
LJ3.2	Luoji Mountain	27.58	102.39	5.39	11.75	0.79	39.44	1119	16.30	1734	southern region
LJ3.3	Luoji Mountain	27.58	102.39	5.37	21.70	0.90	39.44	1119	16.30	1197	southern region
LJ4.1	Luoji Mountain	27.58	102.40	5.67	7.82	0.45	16.34	1119	16.30	2000	southern region
LJ4.2	Luoji Mountain	27.58	102.40	4.21	7.72	0.71	16.34	1119	16.30	2018	southern region
LJ4.3	Luoji Mountain	27.58	102.40	4.25	8.64	0.82	16.34	1119	16.30	1968	southern region
LJ5.1	Luoji Mountain	27.57	102.37	4.04	37.21	1.29	56.85	1117	16.28	1055	southern region
LJ5.2	Luoji Mountain	27.57	102.37	4.17	42.63	1.36	56.85	1117	16.28	1793	southern region
LJ5.3	Luoji Mountain	27.57	102.37	4.48	34.33	1.03	56.85	1117	16.28	1811	southern region
LJ6.1	Luoji Mountain	27.57	102.37	5.65	25.36	1.09	43.39	1117	16.29	1377	southern region
LJ6.2	Luoji Mountain	27.57	102.37	5.73	22.00	1.90	43.39	1117	16.29	1612	southern region
LJ6.3	Luoji Mountain	27.57	102.37	5.80	12.47	0.53	43.39	1117	16.29	1017	southern region
LJ7.1	Luoji Mountain	27.57	102.42	4.44	5.82	0.12	22.08	1119	16.32	2365	southern region
LJ7.2	Luoji Mountain	27.57	102.42	4.75	8.54	0.12	22.08	1119	16.32	2328	southern region
LJ7.3	Luoji Mountain	27.57	102.42	4.15	6.29	0.13	22.08	1119	16.32	2418	southern region
LQ1.1	Longquan Mountain	27.97	119.14	6.45	3.57	0.06	15.91	2214	18.41	1864	southern region
LQ1.2	Longquan Mountain	27.97	119.14	6.30	5.49	0.10	15.91	2214	18.41	1910	southern region
LQ1.3	Longquan Mountain	27.97	119.14	5.91	3.00	0.09	15.91	2214	18.41	1872	southern region
LQ2.1	Longquan Mountain	27.93	119.19	5.88	1.16	0.04	10.95	2210	18.44	1899	southern region
LQ2.2	Longquan Mountain	27.93	119.19	6.14	1.19	0.04	10.95	2210	18.44	1795	southern region
LQ3.1	Longquan Mountain	27.92	119.21	5.87	5.99	0.08	20.74	2208	18.45	1502	southern region
LQ3.2	Longquan Mountain	27.92	119.21	5.47	6.41	0.09	20.74	2208	18.45	1484	southern region
LQ4.1	Longquan Mountain	27.90	119.20	5.25	11.28	0.46	23.81	2209	18.46	1624	southern region
LQ4.2	Longquan Mountain	27.90	119.20	5.26	3.57	0.01	23.81	2209	18.46	1392	southern region
LQ4.3	Longquan Mountain	27.90	119.20	5.36	17.52	0.10	23.81	2209	18.46	1800	southern region
LQ5.1	Longquan Mountain	27.89	119.18	5.28	5.86	0.09	25.75	2210	18.48	1555	southern region
LQ5.3	Longquan Mountain	27.89	119.18	5.64	2.97	0.14	25.75	2210	18.48	1409	southern region
LQ6.2	Longquan Mountain	27.87	119.19	5.30	6.15	0.06	49.81	2210	18.49	1388	southern region
LQ7.1	Longquan Mountain	27.89	119.19	5.12	3.50	0.06	23.81	2209	18.47	1447	southern region
LQ7.2	Longquan Mountain	27.89	119.19	5.05	4.71	0.09	23.81	2209	18.47	1389	southern region
LQ7.3	Longquan Mountain	27.89	119.19	6.18	3.55	0.02	23.81	2209	18.47	1460	southern region
NL1.1	Nanling	24.91	113.01	5.13	12.24	0.40	30.91	2082	19.49	1824	southern region
NL1.2	Nanling	24.91	113.01	5.31	9.21	0.26	30.91	2082	19.49	1955	southern region
NL1.3	Nanling	24.91	113.01	5.35	6.14	0.24	30.91	2082	19.49	1993	southern region
NL2.1	Nanling	24.91	113.02	5.01	13.07	0.17	34.13	2082	19.49	2244	southern region
NL2.2	Nanling	24.91	113.02	5.05	13.76	0.20	34.13	2082	19.49	2103	southern region
NL2.3	Nanling	24.91	113.02	5.04	3.76	0.37	34.13	2082	19.49	2035	southern region
NL3.1	Nanling	24.95	112.99	4.69	15.09	0.48	52.24	2080	19.45	1627	southern region

NL3.2	Nanling	24.95	112.99	4.73	9.82	0.21	52.24	2080	19.45	1795	southern region
NL3.3	Nanling	24.95	112.99	4.69	8.28	0.31	52.24	2080	19.45	1929	southern region
NL4.1	Nanling	24.92	113.01	4.96	13.63	0.15	30.35	2082	19.48	1618	southern region
NL4.2	Nanling	24.92	113.01	5.10	5.26	0.08	30.35	2082	19.48	1919	southern region
NL4.3	Nanling	24.92	113.01	5.19	6.62	0.07	30.35	2082	19.48	1915	southern region
NL5.1	Nanling	24.90	113.03	5.10	15.40	0.14	36.94	2082	19.51	1684	southern region
NL5.2	Nanling	24.90	113.03	5.31	10.85	0.08	36.94	2082	19.51	1813	southern region
NL5.3	Nanling	24.90	113.03	5.27	7.01	0.02	36.94	2082	19.51	1614	southern region
NL6.1	Nanling	24.90	113.05	5.20	7.77	0.11	29.00	2083	19.50	1914	southern region
NL6.2	Nanling	24.90	113.05	4.95	4.66	0.02	29.00	2083	19.50	1379	southern region
NL6.3	Nanling	24.90	113.05	4.73	5.17	0.01	29.00	2083	19.50	1480	southern region
QF1.1	Qingfeng Mountain	34.00	107.44	6.56	13.96	0.97	51.87	706	13.59	2150	northern region
QF1.2	Qingfeng Mountain	34.00	107.44	6.45	9.21	0.87	51.87	706	13.59	2201	northern region
QF1.3	Qingfeng Mountain	34.00	107.44	6.33	17.44	1.08	51.87	706	13.59	2232	northern region
QF2.1	Qingfeng Mountain	34.01	107.44	6.28	10.14	0.20	40.77	704	13.58	2358	northern region
QF2.2	Qingfeng Mountain	34.01	107.44	6.31	12.11	1.28	40.77	704	13.58	2278	northern region
QF2.3	Qingfeng Mountain	34.01	107.44	6.30	9.49	0.26	40.77	704	13.58	2303	northern region
QF3.1	Qingfeng Mountain	34.04	107.44	6.44	9.40	0.79	41.70	699	13.53	1834	northern region
QF3.2	Qingfeng Mountain	34.04	107.44	6.27	20.36	0.34	41.70	699	13.53	2183	northern region
QF3.3	Qingfeng Mountain	34.04	107.44	6.41	13.10	0.31	41.70	699	13.53	1992	northern region
QL1.1	Qinling	34.02	107.80	5.11	6.85	0.47	44.99	696	13.71	2392	northern region
QL1.2	Qinling	34.02	107.80	5.36	10.23	1.26	44.99	696	13.71	2136	northern region
QL1.3	Qinling	34.02	107.80	5.25	5.68	1.88	44.99	696	13.71	2263	northern region
QL10.1	Qinling	34.04	107.61	6.55	8.09	0.51	32.82	696	13.60	2414	northern region
QL10.2	Qinling	34.04	107.61	6.53	13.71	0.10	32.82	696	13.60	2333	northern region
QL10.3	Qinling	34.04	107.61	6.82	6.67	0.87	32.82	696	13.60	2384	northern region
QL2.1	Qinling	34.02	107.79	5.49	3.66	1.12	50.12	695	13.70	2078	northern region
QL2.2	Qinling	34.02	107.79	5.64	12.52	0.94	50.12	695	13.70	2189	northern region
QL2.3	Qinling	34.02	107.79	5.62	14.79	0.92	50.12	695	13.70	2146	northern region
QL3.1	Qinling	34.04	107.79	5.44	10.78	0.67	47.76	692	13.68	2339	northern region
QL3.2	Qinling	34.04	107.79	4.72	10.53	0.97	47.76	692	13.68	2419	northern region
QL3.3	Qinling	34.04	107.79	5.77	15.74	0.51	47.76	692	13.68	2434	northern region
QL4.1	Qinling	34.06	107.79	5.81	12.32	0.96	38.56	689	13.64	2412	northern region
QL4.2	Qinling	34.06	107.79	5.83	12.23	1.00	38.56	689	13.64	2369	northern region
QL4.3	Qinling	34.06	107.79	5.71	20.61	3.78	38.56	689	13.64	2308	northern region
QL6.1	Qinling	34.06	107.76	5.76	18.83	0.69	45.42	690	13.63	2245	northern region
QL6.2	Qinling	34.06	107.76	5.83	12.15	0.77	45.42	690	13.63	2214	northern region
QL6.3	Qinling	34.06	107.76	5.85	14.22	1.53	45.42	690	13.63	2296	northern region

QL7.1	Qinling	34.06	107.76	5.95	13.54	0.82	38.94	690	13.63	2434	northern region
QL7.2	Qinling	34.06	107.76	5.96	9.40	0.55	38.94	690	13.63	2353	northern region
QL7.3	Qinling	34.06	107.76	6.13	13.69	0.90	38.94	690	13.63	2309	northern region
QL9.1	Qinling	34.16	107.65	6.52	6.03	0.40	26.68	674	13.41	2269	northern region
QL9.2	Qinling	34.16	107.65	6.58	7.27	0.26	26.68	674	13.41	2244	northern region
QL9.3	Qinling	34.16	107.65	6.56	8.93	0.26	26.68	674	13.41	2264	northern region
SHB1.1	Saihanba	42.47	117.29	5.52	4.68	0.36	12.12	530	3.98	2014	northern region
SHB1.2	Saihanba	42.47	117.29	5.66	5.75	0.40	12.12	530	3.98	1971	northern region
SHB1.3	Saihanba	42.47	117.29	5.87	5.27	0.28	12.12	530	3.98	2034	northern region
SHB2.1	Saihanba	42.44	117.51	5.74	9.87	0.72	25.67	537	4.36	2129	northern region
SHB2.2	Saihanba	42.44	117.51	5.80	5.33	0.34	25.67	537	4.36	2155	northern region
SHB2.3	Saihanba	42.44	117.51	5.57	13.36	0.79	25.67	537	4.36	2119	northern region
SHB3.1	Saihanba	42.35	117.41	5.70	14.39	1.11	25.92	539	4.36	2085	northern region
SHB3.2	Saihanba	42.35	117.41	5.77	8.92	0.90	25.92	539	4.36	1711	northern region
SHB3.3	Saihanba	42.35	117.41	5.88	11.16	1.01	25.92	539	4.36	2064	northern region
SHB4.1	Saihanba	42.33	117.48	6.00	7.44	0.56	33.48	541	4.48	1971	northern region
SHB4.2	Saihanba	42.33	117.48	5.97	5.80	0.61	33.48	541	4.48	2008	northern region
SHB4.3	Saihanba	42.33	117.48	5.94	8.30	0.60	33.48	541	4.48	2051	northern region
SHWL1.1	Saihanwula	44.19	118.71	5.99	10.75	1.00	19.97	509	3.98	2009	northern region
SHWL1.2	Saihanwula	44.19	118.71	6.08	8.49	0.21	19.97	509	3.98	2045	northern region
SHWL1.3	Saihanwula	44.19	118.71	6.27	8.31	0.64	19.97	509	3.98	2113	northern region
SHWL2.1	Saihanwula	44.20	118.72	5.62	6.25	0.48	15.45	509	3.98	1766	northern region
SHWL2.2	Saihanwula	44.20	118.72	5.56	4.69	0.43	15.45	509	3.98	1881	northern region
SHWL2.3	Saihanwula	44.20	118.72	5.32	3.54	0.68	15.45	509	3.98	1559	northern region
SHWL3.1	Saihanwula	44.21	118.72	5.73	2.91	0.26	17.70	509	3.95	1754	northern region
SHWL3.3	Saihanwula	44.21	118.72	5.70	8.75	0.56	17.70	509	3.95	2028	northern region
SHWL4.1	Saihanwula	44.27	118.41	6.00	3.25	0.32	19.17	502	3.34	2128	northern region
SHWL4.2	Saihanwula	44.27	118.41	6.13	2.33	0.22	19.17	502	3.34	2026	northern region
SHWL4.3	Saihanwula	44.27	118.41	6.15	4.77	0.42	19.17	502	3.34	1960	northern region
SHWL5.1	Saihanwula	44.27	118.42	6.20	3.63	0.28	21.25	502	3.34	2133	northern region
SHWL5.2	Saihanwula	44.27	118.42	6.27	3.45	0.32	21.25	502	3.34	2159	northern region
SHWL5.3	Saihanwula	44.27	118.42	6.29	4.30	0.37	21.25	502	3.34	2145	northern region
SHWL6.1	Saihanwula	44.27	118.43	6.31	3.47	0.25	19.65	503	3.36	1935	northern region
SHWL6.2	Saihanwula	44.27	118.43	6.35	3.50	0.40	19.65	503	3.36	1876	northern region
SHWL6.3	Saihanwula	44.27	118.43	6.32	2.83	0.28	19.65	503	3.36	1619	northern region
SNJ1.1	Shennong Jia	31.45	110.40	5.82	19.75	1.09	44.52	1097	16.01	2285	northern region
SNJ1.2	Shennong Jia	31.45	110.40	5.82	19.72	1.10	44.52	1097	16.01	2339	northern region
SNJ1.3	Shennong Jia	31.45	110.40	5.82	19.74	1.11	44.52	1097	16.01	2295	northern region

SNJ10.2	Shennong Jia	31.67	110.42	5.73	22.47	1.45	27.06	1052	15.91	2329	northern region
SNJ10.3	Shennong Jia	31.67	110.42	5.73	22.47	1.43	27.06	1052	15.91	2277	northern region
SNJ11.1	Shennong Jia	31.76	110.49	6.03	11.32	0.69	39.56	1030	15.88	2163	northern region
SNJ11.2	Shennong Jia	31.76	110.49	6.03	11.32	0.60	39.56	1030	15.88	2230	northern region
SNJ11.3	Shennong Jia	31.76	110.49	6.03	11.32	0.58	39.56	1030	15.88	2118	northern region
SNJ12.1	Shennong Jia	31.49	110.31	5.70	9.98	0.65	44.68	1095	15.97	2156	northern region
SNJ12.2	Shennong Jia	31.49	110.31	5.70	9.98	0.69	44.68	1095	15.97	2129	northern region
SNJ12.3	Shennong Jia	31.49	110.31	5.70	9.98	0.81	44.68	1095	15.97	2169	northern region
SNJ13.1	Shennong Jia	31.46	110.28	4.88	16.75	1.53	43.28	1102	15.98	2173	northern region
SNJ13.2	Shennong Jia	31.46	110.28	4.88	16.75	1.41	43.28	1102	15.98	2210	northern region
SNJ13.3	Shennong Jia	31.46	110.28	4.88	16.75	1.52	43.28	1102	15.98	2133	northern region
SNJ14.1	Shennong Jia	31.44	110.30	4.98	8.42	0.54	43.12	1105	15.99	1567	northern region
SNJ14.2	Shennong Jia	31.44	110.30	4.98	8.42	0.50	43.12	1105	15.99	1520	northern region
SNJ14.3	Shennong Jia	31.44	110.30	4.98	8.42	0.67	43.12	1105	15.99	1545	northern region
SNJ15.1	Shennong Jia	31.44	110.31	5.22	14.64	1.10	57.66	1105	15.99	1908	northern region
SNJ15.2	Shennong Jia	31.44	110.31	5.22	14.64	1.22	57.66	1105	15.99	1054	northern region
SNJ15.3	Shennong Jia	31.44	110.31	5.22	14.64	1.14	57.66	1105	15.99	1036	northern region
SNJ2.1	Shennong Jia	31.48	110.37	5.58	4.32	0.31	57.16	1093	15.99	2268	northern region
SNJ2.2	Shennong Jia	31.48	110.37	5.58	4.21	0.37	57.16	1093	15.99	1967	northern region
SNJ2.3	Shennong Jia	31.48	110.37	5.58	4.26	0.26	57.16	1093	15.99	1982	northern region
SNJ3.1	Shennong Jia	31.45	110.15	5.99	17.91	0.80	54.15	1111	15.94	2178	northern region
SNJ3.2	Shennong Jia	31.45	110.15	5.99	18.30	0.80	54.15	1111	15.94	2174	northern region
SNJ3.3	Shennong Jia	31.45	110.15	5.99	18.11	0.95	54.15	1111	15.94	2203	northern region
SNJ4.1	Shennong Jia	31.47	110.15	5.83	9.91	0.64	49.12	1107	15.94	1908	northern region
SNJ4.2	Shennong Jia	31.47	110.15	5.83	10.49	0.63	49.12	1107	15.94	1982	northern region
SNJ4.3	Shennong Jia	31.47	110.15	5.83	10.20	0.61	49.12	1107	15.94	1949	northern region
SNJ5.1	Shennong Jia	31.45	110.19	5.82	7.83	0.85	48.09	1110	15.96	1888	northern region
SNJ5.2	Shennong Jia	31.45	110.19	5.82	7.83	1.12	48.09	1110	15.96	1854	northern region
SNJ5.3	Shennong Jia	31.45	110.19	5.82	7.83	1.23	48.09	1110	15.96	1896	northern region
SNJ6.1	Shennong Jia	31.74	110.74	5.80	6.37	0.75	35.72	1021	15.95	2442	northern region
SNJ6.2	Shennong Jia	31.74	110.74	5.80	5.63	0.72	35.72	1021	15.95	2376	northern region
SNJ6.3	Shennong Jia	31.74	110.74	5.80	6.00	0.65	35.72	1021	15.95	2364	northern region
SNJ7.1	Shennong Jia	31.76	110.55	5.77	17.75	1.26	25.10	1027	15.89	2325	northern region
SNJ7.2	Shennong Jia	31.76	110.55	5.77	17.62	1.20	25.10	1027	15.89	2290	northern region
SNJ7.3	Shennong Jia	31.76	110.55	5.77	17.69	0.80	25.10	1027	15.89	2328	northern region
SNJ8.1	Shennong Jia	31.61	110.42	5.92	11.41	0.57	26.17	1064	15.94	2216	northern region
SNJ8.2	Shennong Jia	31.61	110.42	5.92	11.41	0.58	26.17	1064	15.94	2186	northern region
SNJ8.3	Shennong Jia	31.61	110.42	5.92	11.41	0.97	26.17	1064	15.94	2219	northern region

SNJ9.1	Shennong Jia	31.76	110.54	5.83	4.64	0.43	26.55	1028	15.89	2136	northern region
SNJ9.2	Shennong Jia	31.76	110.54	5.83	4.64	0.42	26.55	1028	15.89	2178	northern region
SNJ9.3	Shennong Jia	31.76	110.54	5.83	4.64	0.45	26.55	1028	15.89	2138	northern region
SWD1.1	Shiwanda Mountain	21.88	107.91	4.13	7.25	0.30	38.21	1822	22.75	1284	southern region
SWD1.2	Shiwanda Mountain	21.88	107.91	4.13	7.25	0.28	38.21	1822	22.75	1360	southern region
SWD1.3	Shiwanda Mountain	21.88	107.91	4.13	7.25	0.30	38.21	1822	22.75	1412	southern region
SWD2.1	Shiwanda Mountain	21.88	107.92	4.52	6.96	0.12	46.11	1824	22.76	1759	southern region
SWD2.2	Shiwanda Mountain	21.88	107.92	4.52	6.96	0.13	46.11	1824	22.76	1842	southern region
SWD2.3	Shiwanda Mountain	21.88	107.92	4.52	6.96	0.16	46.11	1824	22.76	1337	southern region
SWD3.1	Shiwanda Mountain	21.89	107.91	4.80	6.90	0.19	22.65	1821	22.75	1415	southern region
SWD3.2	Shiwanda Mountain	21.89	107.91	4.80	6.90	0.16	22.65	1821	22.75	1492	southern region
SWD3.3	Shiwanda Mountain	21.89	107.91	4.80	6.90	0.13	22.65	1821	22.75	1386	southern region
SWD4.1	Shiwanda Mountain	21.90	107.91	4.48	5.66	0.14	15.49	1819	22.74	1226	southern region
SWD4.2	Shiwanda Mountain	21.90	107.91	4.48	5.66	0.14	15.49	1819	22.74	1266	southern region
SWD4.3	Shiwanda Mountain	21.90	107.91	4.48	5.66	0.29	15.49	1819	22.74	1192	southern region
SWD5.1	Shiwanda Mountain	21.90	107.90	4.05	4.56	0.23	34.81	1819	22.74	1128	southern region
SWD5.2	Shiwanda Mountain	21.90	107.90	4.05	4.56	0.21	34.81	1819	22.74	1222	southern region
SWD5.3	Shiwanda Mountain	21.90	107.90	4.05	4.56	0.18	34.81	1819	22.74	1190	southern region
SYK1.1	Suyukou	38.73	105.91	6.31	6.69	0.20	30.31	299	8.87	2178	northern region
SYK1.2	Suyukou	38.73	105.91	6.35	6.52	0.24	30.31	299	8.87	2232	northern region
SYK2.1	Suyukou	38.74	105.91	6.55	2.41	0.18	5.74	299	8.87	2237	northern region
SYK2.2	Suyukou	38.74	105.91	6.49	1.99	0.16	5.74	299	8.87	2254	northern region
SYK2.3	Suyukou	38.74	105.91	6.54	2.10	0.16	5.74	299	8.87	2348	northern region
SYK3.1	Suyukou	38.74	105.91	6.27	4.67	0.30	25.86	299	8.87	2330	northern region
SYK3.2	Suyukou	38.74	105.91	6.28	5.52	0.29	25.86	299	8.87	2395	northern region
SYK3.3	Suyukou	38.74	105.91	6.33	6.76	0.30	25.86	299	8.87	2301	northern region
SYK4.1	Suyukou	38.75	105.92	6.42	4.54	0.30	31.34	298	8.87	2264	northern region
SYK4.2	Suyukou	38.75	105.92	6.50	5.54	0.32	31.34	298	8.87	2439	northern region
SYK4.3	Suyukou	38.75	105.92	6.39	5.50	0.35	31.34	298	8.87	2313	northern region
TM1.1	Tianmu Mountain	30.58	119.71	4.35	7.72	0.09	20.17	1692	16.87	1325	southern region
TM1.2	Tianmu Mountain	30.58	119.71	4.51	7.55	0.04	20.17	1692	16.87	1480	southern region
TM1.3	Tianmu Mountain	30.58	119.71	4.92	7.46	0.13	20.17	1692	16.87	2000	southern region
TM2.1	Tianmu Mountain	30.57	119.73	4.56	4.26	0.37	32.94	1696	16.87	1571	southern region
TM2.2	Tianmu Mountain	30.57	119.73	4.20	5.72	0.57	32.94	1696	16.87	1968	southern region
TM2.3	Tianmu Mountain	30.57	119.73	4.38	4.33	0.37	32.94	1696	16.87	1367	southern region
TM3.1	Tianmu Mountain	30.36	119.43	4.51	4.39	0.36	41.38	1766	16.97	1368	southern region
TM3.3	Tianmu Mountain	30.36	119.43	4.75	2.86	0.26	41.38	1766	16.97	1808	southern region
TM4.2	Tianmu Mountain	30.34	119.46	6.20	2.39	0.26	43.04	1774	16.99	1873	southern region

TM5.1	Tianmu Mountain	30.31	119.49	6.17	2.99	0.34	36.76	1784	17.00	1957	southern region
TM5.2	Tianmu Mountain	30.31	119.49	6.08	2.54	0.26	36.76	1784	17.00	1871	southern region
WG1.1	Wugong Mountain	27.46	114.16	5.91	4.72	0.14	37.31	2067	18.17	1526	southern region
WG1.2	Wugong Mountain	27.46	114.16	6.25	2.77	0.03	37.31	2067	18.17	1417	southern region
WG1.3	Wugong Mountain	27.46	114.16	6.06	2.50	0.04	37.31	2067	18.17	1623	southern region
WG2.1	Wugong Mountain	27.46	114.17	4.97	12.38	0.08	45.63	2068	18.17	1591	southern region
WG2.2	Wugong Mountain	27.46	114.17	5.41	14.54	0.10	45.63	2068	18.17	1680	southern region
WG2.3	Wugong Mountain	27.46	114.17	5.51	12.03	0.14	45.63	2068	18.17	1498	southern region
WG3.1	Wugong Mountain	27.46	114.17	5.10	15.05	0.13	54.29	2068	18.17	1898	southern region
WG3.2	Wugong Mountain	27.46	114.17	4.88	11.81	0.13	54.29	2068	18.17	1894	southern region
WG3.3	Wugong Mountain	27.46	114.17	5.38	9.83	0.09	54.29	2068	18.17	1883	southern region
WG4.1	Wugong Mountain	27.46	114.17	5.60	4.76	0.07	41.19	2068	18.17	1604	southern region
WG4.2	Wugong Mountain	27.46	114.17	4.87	5.19	0.06	41.19	2068	18.17	1555	southern region
WG4.3	Wugong Mountain	27.46	114.17	5.97	4.91	0.07	41.19	2068	18.17	1464	southern region
WG5.1	Wugong Mountain	27.47	114.17	5.70	7.20	0.05	34.77	2068	18.17	1396	southern region
WG5.2	Wugong Mountain	27.47	114.17	5.64	5.20	0.05	34.77	2068	18.17	1588	southern region
WG5.3	Wugong Mountain	27.47	114.17	5.41	4.43	0.05	34.77	2068	18.17	1479	southern region
WYZ1.1	Wuyuezhai	38.72	113.84	5.31	4.20	0.27	31.28	525	11.07	1874	northern region
WYZ1.2	Wuyuezhai	38.72	113.84	5.82	3.60	0.24	31.28	525	11.07	1966	northern region
WYZ1.3	Wuyuezhai	38.72	113.84	5.74	3.59	0.28	31.28	525	11.07	2027	northern region
WYZ2.1	Wuyuezhai	38.72	113.84	5.76	3.66	0.32	31.31	525	11.08	2074	northern region
WYZ2.2	Wuyuezhai	38.72	113.84	5.50	4.85	0.38	31.31	525	11.08	2138	northern region
WYZ2.3	Wuyuezhai	38.72	113.84	5.42	4.53	0.37	31.31	525	11.08	1989	northern region
WYZ3.1	Wuyuezhai	38.72	113.84	5.67	4.50	0.42	52.51	525	11.08	2038	northern region
WYZ3.2	Wuyuezhai	38.72	113.84	5.63	5.64	0.43	52.51	525	11.08	1987	northern region
WYZ3.3	Wuyuezhai	38.72	113.84	5.72	5.30	0.42	52.51	525	11.08	2051	northern region
WYZ4.1	Wuyuezhai	38.73	113.85	5.82	2.86	0.30	24.65	525	11.06	2227	northern region
WYZ4.2	Wuyuezhai	38.73	113.85	6.10	4.91	0.43	24.65	525	11.06	2350	northern region
WYZ4.3	Wuyuezhai	38.73	113.85	6.20	4.96	0.38	24.65	525	11.06	2257	northern region
WYZ5.1	Wuyuezhai	38.72	113.86	5.45	4.25	0.19	34.25	526	11.09	2174	northern region
WYZ5.2	Wuyuezhai	38.72	113.86	5.65	3.15	0.20	34.25	526	11.09	2297	northern region
WYZ5.3	Wuyuezhai	38.72	113.86	5.67	2.77	0.14	34.25	526	11.09	2132	northern region
XX1.1	Xiaoxing'anling	47.45	129.65	7.05	10.83	1.05	37.55	665	2.32	2069	northern region
XX1.2	Xiaoxing'anling	47.45	129.65	6.93	10.29	0.91	37.55	665	2.32	2029	northern region
XX1.3	Xiaoxing'anling	47.45	129.65	6.91	12.10	1.05	37.55	665	2.32	1945	northern region
XX10.1	Xiaoxing'anling	46.63	128.47	4.54	6.29	0.60	36.07	693	2.79	1424	northern region
XX10.2	Xiaoxing'anling	46.63	128.47	4.70	8.06	0.68	36.07	693	2.79	1857	northern region
XX10.3	Xiaoxing'anling	46.63	128.47	4.75	9.04	0.69	36.07	693	2.79	1647	northern region

XX2.1	Xiaoxing'anling	47.60	129.20	6.44	10.09	1.07	23.34	664	2.16	2096	northern region
XX2.3	Xiaoxing'anling	47.60	129.20	6.22	9.69	0.80	23.34	664	2.16	1813	northern region
XX3.1	Xiaoxing'anling	48.24	129.57	6.07	18.55	1.18	40.40	640	1.69	2144	northern region
XX3.2	Xiaoxing'anling	48.24	129.57	5.99	23.84	0.90	40.40	640	1.69	2085	northern region
XX4.1	Xiaoxing'anling	48.48	128.99	6.20	12.67	0.98	37.26	635	1.42	1823	northern region
XX4.2	Xiaoxing'anling	48.48	128.99	6.61	13.72	0.83	37.26	635	1.42	1406	northern region
XX4.3	Xiaoxing'anling	48.48	128.99	6.71	12.40	0.92	37.26	635	1.42	1814	northern region
XX5.1	Xiaoxing'anling	48.85	128.91	6.41	8.60	0.66	23.64	619	1.10	2102	northern region
XX5.2	Xiaoxing'anling	48.85	128.91	6.10	15.08	0.45	23.64	619	1.10	1761	northern region
XX5.3	Xiaoxing'anling	48.85	128.91	5.47	9.54	0.67	23.64	619	1.10	1884	northern region
XX6.1	Xiaoxing'anling	46.63	128.54	6.01	15.91	0.94	24.54	692	2.78	2013	northern region
XX6.2	Xiaoxing'anling	46.63	128.54	5.99	13.80	1.11	24.54	692	2.78	1718	northern region
XX6.3	Xiaoxing'anling	46.63	128.54	6.12	6.11	0.51	24.54	692	2.78	1972	northern region
XX7.1	Xiaoxing'anling	46.63	128.52	5.90	20.78	1.30	33.29	692	2.78	2383	northern region
XX7.2	Xiaoxing'anling	46.63	128.52	5.97	21.12	0.64	33.29	692	2.78	1868	northern region
XX7.3	Xiaoxing'anling	46.63	128.52	5.98	15.72	0.95	33.29	692	2.78	1998	northern region
XX8.1	Xiaoxing'anling	46.64	128.51	5.64	4.94	0.46	30.57	692	2.78	1669	northern region
XX8.2	Xiaoxing'anling	46.64	128.51	5.54	8.20	0.55	30.57	692	2.78	1622	northern region
XX8.3	Xiaoxing'anling	46.64	128.51	5.44	5.38	0.57	30.57	692	2.78	1566	northern region
XX9.1	Xiaoxing'anling	46.63	128.49	5.01	10.85	0.71	35.01	692	2.78	1627	northern region
XX9.2	Xiaoxing'anling	46.63	128.49	4.91	10.47	0.82	35.01	692	2.78	1574	northern region
XX9.3	Xiaoxing'anling	46.63	128.49	4.74	10.12	0.73	35.01	692	2.78	1604	northern region
