# Supplementary material for: Resistance potential of soil bacterial communities along a biodiversity gradient in forest ecosystems
Source: mLife. 2022 Nov 3;1(4):399–411. doi: 10.1002/mlf2.12042 (PMC10989803; doi:10.1002/mlf2.12042)
Supplement: Supplementary file 3 — Supporting information. [file MLF2-1-399-s002.pdf]

Table S2. Pearson correlations (*r*) between the values of CMTB (or CMRA) that were calculated by different approaches of bacterial OTUs selection at different levels. For the approach based on a consistent abundance of bacterial communities, the bacterial OTUs of a sample were ranked by their relative abundances in descending order. The most abundant OTUs, representing the accumulated abundances at 0.75, 0.80, 0.85, and 0.9, respectively, were kept for the index calculation. For the approach based on a consistent number of bacterial richness, 300, 500, 700, and 900 bacterial OTUs of a sample were randomly selected, respectively, and the OTUs selection at each level was repeated 100 times. For each level, the indexes were calculated as the average values of these 100 sets of bacterial OTUs.

CMTB		Abundance				Random			
		0.75	0.8	0.85	0.9	300	500	700	900
Abundance	0.75	1.0000							
	0.8	0.9998	1.0000						
	0.85	0.9995	0.9998	1.0000					
	0.9	0.9991	0.9995	0.9998	1.0000				
Random	300	0.9878	0.9893	0.9905	0.9913	1.0000			
	500	0.9927	0.9940	0.9949	0.9955	0.9980	1.0000		
	700	0.9949	0.9959	0.9967	0.9972	0.9972	0.9990	1.0000	
	900	0.9959	0.9969	0.9976	0.9981	0.9959	0.9986	0.9992	1.0000

CMRA		Abundance				Random			
		0.75	0.8	0.85	0.9	300	500	700	900
Abundance	0.75	1.0000							
	0.8	0.9992	1.0000						
	0.85	0.9974	0.9993	1.0000					
	0.9	0.9950	0.9976	0.9993	1.0000				
Random	300	0.9687	0.9738	0.9789	0.9837	1.0000			
	500	0.9777	0.9822	0.9866	0.9906	0.9961	1.0000		
	700	0.9821	0.9861	0.9901	0.9936	0.9949	0.9973	1.0000	
	900	0.9843	0.9883	0.9922	0.9955	0.9932	0.9969	0.9983	1.0000
